# The role of ATXR6 expression in modulating genome stability and transposable element repression in *Arabidopsis*

**DOI:** 10.1073/pnas.2115570119

**Published:** 2022-01-13

**Authors:** Magdalena E. Potok, Zhenhui Zhong, Colette L. Picard, Qikun Liu, Truman Do, Cassidy E. Jacobsen, Ocean Sakr, Bilguudei Naranbaatar, Ruwan Thilakaratne, Zhanna Khnkoyan, Megan Purl, Harrison Cheng, Helena Vervaet, Suhua Feng, Shima Rayatpisheh, James A. Wohlschlegel, Ronan C. O’Malley, Joseph R. Ecker, Steven E. Jacobsen

**Affiliations:** ^a^Department of Molecular, Cell and Developmental Biology, University of California, Los Angeles, CA 90095;; ^b^School of Advanced Agricultural Sciences, Peking University, 100871 Beijing, China;; ^c^Department of Biological Chemistry, David Geffen School of Medicine, University of California, Los Angeles, CA 90095;; ^d^HHMI, The Salk Institute for Biological Studies, La Jolla, CA 92037;; ^e^Plant Biology Laboratory, The Salk Institute for Biological Studies, La Jolla, CA 92037;; ^f^HHMI, University of California, Los Angeles, CA 90095

**Keywords:** H3K27me1, ATXR5/6, plant, histone methyltransferase

## Abstract

ARABIDOPSIS TRITHORAX-RELATED PROTEIN 5 (ATXR5) AND ATXR6 are required for the deposition of H3K27me1 and for maintaining genomic stability in *Arabidopsis*. Reduction of ATXR5/6 activity results in activation of DNA damage response genes, along with tissue-specific derepression of transposable elements (TEs), chromocenter decompaction, and genomic instability characterized by accumulation of excess DNA from heterochromatin. How loss of ATXR5/6 and H3K27me1 leads to these phenotypes remains unclear. Here we provide extensive characterization of the *atxr5/6* hypomorphic mutant by comprehensively examining gene expression and epigenetic changes in the mutant. We found that the tissue-specific phenotypes of TE derepression and excessive DNA in this *atxr5/6* mutant correlated with residual *ATXR6* expression from the hypomorphic *ATXR6* allele. However, up-regulation of DNA damage genes occurred regardless of *ATXR6* levels and thus appears to be a separable process. We also isolated an *atxr6*-null allele which showed that ATXR5 and ATXR6 are required for female germline development. Finally, we characterize three previously reported suppressors of the hypomorphic *atxr5/6* mutant and show that these rescue *atxr5/6* via distinct mechanisms, two of which involve increasing H3K27me1 levels.

## Significance

The plant-specific H3K27me1 methyltransferases ATXR5 and ATXR6 play integral roles connecting epigenetic silencing with genomic stability. However, how H3K27me1 relates to these processes is poorly understood. In this study, we performed a comprehensive transcriptome analysis of tissue- and ploidy-specific expression in a hypomorphic *atxr5/6* mutant and revealed that the tissue-specific defects correlate with residual *ATXR6* expression. We also determined that ATXR5/6 function is essential for female germline development. Furthermore, we provide a comprehensive analysis of H3K27me1 changes in relation to other epigenetic marks. We also determined that some previously reported suppressors of *atxr5/6* may act by restoring the levels of H3K27me1, such as through up-regulation of the *ATXR6* transcript in the *atxr6* hypomorphic promoter allele.

Faithful duplication of genetic material is essential for the maintenance of genome stability in eukaryotes. This requires a coordination of chromatin changes and RNA transcription and DNA replication processes during cell division. ARABIDOPSIS TRITHORAX-RELATED PROTEIN 5 (ATXR5) and ATXR6 encode redundant histone methyltransferases that deposit histone H3 lysine 27 monomethylation (H3K27me1) in *Arabidopsis thaliana* chromatin (1). In interphase nuclei, H3K27me1 is enriched in highly condensed heterochromatic regions consisting of repetitive elements, pericentromeric regions (which can be visualized as chromocenters), and ribosomal genes (1). ATXR5 and ATXR6 specifically deposit H3K27me1 on histone H3.1, and this is thought to occur during S phase (2–4). Chromocenters are also enriched in H3 lysine 9 dimethylation (H3K9me2) and DNA methylation (5–8).

Loss of factors responsible for DNA methylation and H3K27me1 results in decondensation of chromocenters and reactivation of transposable elements (TEs) (1, 9). Although DNA methylation and H3K27me1 cooperate in repressing TEs (10), they do so via independent pathways, since a reduction in H3K27me1 has little effect on DNA methylation (1). Interestingly, depletion of H3K27me1 levels in the hypomorphic *atxr5/6* mutant results in a genomic instability defect characterized by the accumulation of excess DNA corresponding to heterochromatic regions (11). This genomic instability phenotype has only been observed in higher-ploidy cells in leaves and cotyledons that have undergone endoreduplication (12), a modified cell cycle where the genome duplicates without cellular division. Moreover, the chromocenter decondensation phenotype observed in *atxr5/6* is also specific to endoreduplicated cells, and their chromatin forms unique donut-like structures termed repair-associated centers (RACs) as a response to DNA damage in heterochromatin in the mutant (13). Furthermore, *atxr5/6* mutants are characterized by the activation of genes involved in DNA damage repair such as the *Arabidopsis* homologs of human RAD51 and BRCA1 (10). Together, these findings suggest that the H3K27me1 mark is an integral player in the cross-talk between replication, transcription, genome organization, and genome stability. However, how these events are connected and what features underlie the tissue-specific defects is poorly understood.

In this study, we found that the transposon activation and excessive DNA phenotypes of *atxr5/6* hypomorphic mutants inversely correlate with the level of *ATXR6* transcript rather than with endoreduplication. Furthermore, characterization of an *atxr6*-null allele revealed that ATXR5/6 function is essential for female germline development. We also extensively profiled chromatin and transcriptome changes associated with the partial loss of H3K27me1 in the *atxr5/6* hypomorphic mutant. Lastly, we determined that some, but not all, previously isolated genetic suppressors of the *atxr5/6* hypomorphic mutant act by restoring H3K27me1 levels, for instance via the up-regulation of the ATXR6 transcript in the *atxr6* hypomorphic promoter allele.

## Results

### Tissue-Specific Levels of ATXR6 Correlate with the Severity of the *atxr5 atxr6* Mutant Phenotype.

**Fig. 1.**
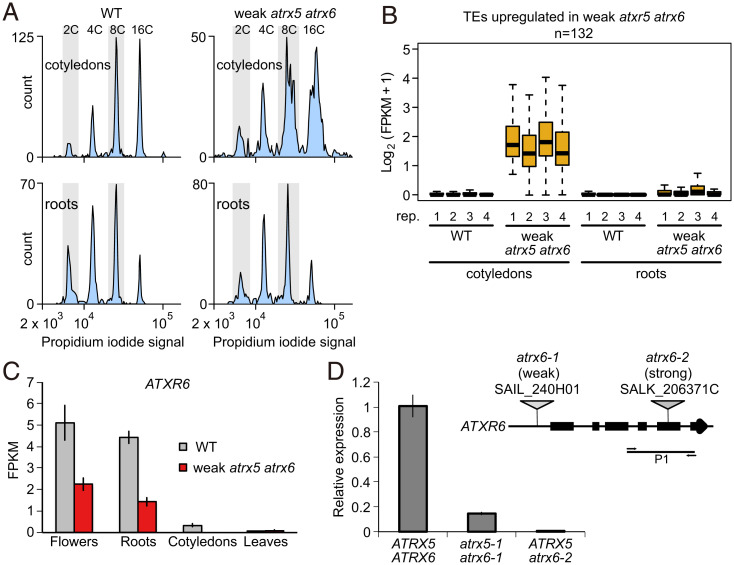
Tissue-specific defects in *atxr5/6* mutants. (*A*) Flow cytometry profiles of nuclei from wild-type (WT) and *atxr5/6* (W) mutant cotyledons and root tissue. (*B*) Boxplot of log2(FPKM + 1) expression values for TEs up-regulated in *atxr5/6* cotyledons, in four replicates each of wild-type and *atxr5/6* cotyledons and roots. Center lines indicate the median, upper and lower bounds represent the 75th and 25th percentiles, respectively, whiskers indicate the minimum and the maximum, and outliers are not shown. (*C*) Bar chart of RNA-seq FPKM values for *ATXR6* expression in wild-type plants and *atxr5/6* (W) mutants in floral (*n* = 3), root (*n* = 4), cotyledon (*n* = 4), and leaf (*n* = 3) tissues. Bars represent average and whiskers represent ± SE (SEM) across the three or four biological replicates. (*D*, *Top*) Genomic structure of *ATXR6* with T-DNA insertion sites indicated. Horizontal line represents the amplified region (P1) for qRT-PCR. (*D*, *Bottom*) qRT-PCR analysis of *ATXR6* expression in 2-wk-old seedlings in wild-type, *atxr5-1 atxr6-1* (W/W), and *ATXR5* *atxr6-2* (S/S) plants. Bar represents average and whiskers represent ± SE (SEM) from *n* = 3 biological replicates of the indicated samples.

We previously observed that the molecular phenotypes of the hypomorphic *atxr5-1 atxr6-1* double mutant [hereafter termed *atxr5/6* weak (W)] were specific to the endoreduplicating cells of mature leaves and cotyledons but not immature floral buds that lack endoreduplication (13), suggesting that defects in the *atxr5/6* (W) mutant may be specific to endoreduplicating tissues. These phenotypes include TE derepression and excess DNA derived from heterochromatic regions, the latter evident during flow cytometry as a characteristic shoulder on peaks corresponding to 8C and 16C nuclei after DAPI staining (11). However, when we repeated this experiment in roots, which also have high levels of endoreduplication, we found that *atxr5/6* (W) roots did not display the excess DNA phenotype, and instead resembled wild-type plants (Fig. 1*A*). We also performed RNA sequencing (RNA-seq) of cotyledons and roots in wild-type and *atxr5/6* (W) plants and found very little TE derepression in *atxr5/6* (W) in roots (Fig. 1*B*), which was further confirmed by qRT-PCR (*SI Appendix*, Fig. S1). Given that the *atxr6-1* allele in *atxr5/6* (W) is a hypomorphic mutation resulting from a T-DNA insertion in the promoter (1), this prompted us to investigate whether the tissue-specific phenotype may correlate better with the levels of residual ATXR6 expression rather than with the levels of endoreduplication. Indeed, tissue-specific RNA-seq analysis in wild-type and *atxr5/6* (W) plants showed that levels of *ATXR6* expression in *atxr5/6* (W) were much higher in roots and flowers but barely detectable in cotyledons and leaves (Fig. 1*C*). This suggests that the *atxr5/6* (W) phenotypes may result from tissue-specific patterns of residual *ATXR6* expression, rather than the level of endoreduplication.

We isolated an *atxr6* allele that carried a T-DNA insertion (SALK_206371) in the coding region of *ATXR6* (*atxr6-2*), hereafter termed *atxr6-2* strong (S) (Fig. 1*D*). *ATXR6* RNA was undetectable near the SALK_206371 insertion site in seedlings carrying the *atxr6-2* (S) allele, whereas reduced expression could be detected in *atxr6-1* (W) seedlings (Fig. 1*D*). Interestingly, we were unable to recover *atxr5-1* (^−/−^) homozygotes from self-pollination of *atxr6-2* (S/S) *atxr5-1* (^+/−^) plants (*n* = 123), nor did we recover *atxr6-2* (S/S) homozygotes from a self-cross of *atxr6-1/atxr6-2* (S/W) *atxr5*-*1* (^−/−^) (*n* = 163), suggesting that combined loss of both *ATXR5* and *ATXR6* is lethal (Table 1). To determine the mode of lethality, we performed reciprocal crosses between *atxr6-1/atxr6-2* (S/W) *atxr5-1* (^−/−^) and wild-type *ATXR5* (^+/+^) *ATXR6* (^+/+^) plants. The transmission efficiency of the *atxr6-1* (S) allele was 51% (*n* = 107) through the male gametophyte, suggesting that male gametogenesis was not affected by loss of both *ATXR5* and *ATXR6* (Table 2). In contrast, we failed to recover any *atxr6-2* (S) alleles (*n* = 116) through the female gametophyte, suggesting that the *atxr6-2* (S) allele causes female gametophytic lethality in the *atxr5-1* mutant background (Table 2). Together, these data indicate that *ATXR5* and *ATXR6* are required for viability, and that in the presence of a weak *ATXR6* allele, molecular phenotypes correlate with varying levels of residual *ATXR6* expression across different tissues.

**Table 1.** Self-pollinations

Parent				
*atxr5-1* (^+/−^) *atxr6-2* (S/S)	*atxr5-1* (^+/+^)	*atxr5-1* (^+/−^)	*atxr5-1* (^−/−^)	Total
	70	53	0	123
*atxr5-1* (^−/−^) *atxr6-1/atxr6-2* (S/W)	*atxr6-1* (W/W)	*atxr6-1/atxr6-2* (S/W)	*atxr6-2* (S/S)	
	81	82	0	163
Total				286

**Table 2.** Reciprocal crosses

Parent	*atxr6-2* (^−/+^)	*atxr6-1* (^−/+^)
*ATXR5* (^+/+^) *ATXR6* (^+/+^) ♀ x *atxr5-1* (^−/−^) *atxr6-1/atxr6-2* (S/W) ♂	55	52
*ATXR5* (^+/+^) *ATXR6* (^+/+^) ♂ x *atxr5-1* (^−/−^) *atxr6-1/atxr6-2* (S/W) ♀	0	116

### Chromatin Changes in the *atxr5/6* (W) Mutant.

Previous immunofluorescence studies reported that chromocenters in *atxr5/6* (W) become decondensed and form hollow ring structures, reflecting a response to DNA damage (13). This phenotype was specifically observed in higher-ploidy, endoreduplicated nuclei in leaves (1, 13), and occurred without major changes in H3K9me2 or DNA methylation levels (1). To better understand the mechanism of chromocenter remodeling in *atxr5/6* (W), we profiled a number of additional chromatin modifications using low-input chromatin immunoprecipitation sequencing (ChIP-seq) of cotyledons, which have low *ATXR6* expression and a high proportion of endoreduplicated and defective nuclei in *atxr5/6* (W) (*SI Appendix*, Fig. S2). Profiles for the different histone modifications over protein-coding genes in cotyledons were consistent with prior published datasets (14), validating our ChIP-seq methods (*SI Appendix*, Fig. S3). As previously reported, H3K27me1 was reduced in pericentromeric heterochromatin in *atxr5/6* (W) compared with wild-type cotyledons (*SI Appendix*, Fig. S4) (14–16). Although we did not observe substantial changes in activating/euchromatin-specific histone modifications (H3K4me3, H3K14Ac, H3Ac, H4Ac, H2A.Z) over pericentromeric heterochromatin regions (*SI Appendix*, Fig. S4), we did detect a moderate increase in H3K4me3, H2A.Z, and histone acetylation marks (H3K27Ac, H4Ac, H3K14Ac, H3Ac) at TEs that were up-regulated in *atxr5/6* (W) (*SI Appendix*, Fig. S5), likely reflecting the transcriptional up-regulation of these regions. Additionally, we observed a slight increase in H3K9me2 over pericentromeric heterochromatin, accompanied by a reduction in H3K4me1 (*SI Appendix*, Fig. S4). Our observations are consistent with recent publications where an increase in H3K9me2 and H3K27Ac marks was observed in the *atxr5/6* (W) mutant (17, 18). Although we cannot rule out that other untested epigenetic marks may be involved, these data suggest that loss of H3K27me1 is the major contributor to the chromocenter decompaction phenotype observed in *atxr5/6* (W) mutants.

**Fig. 2.**
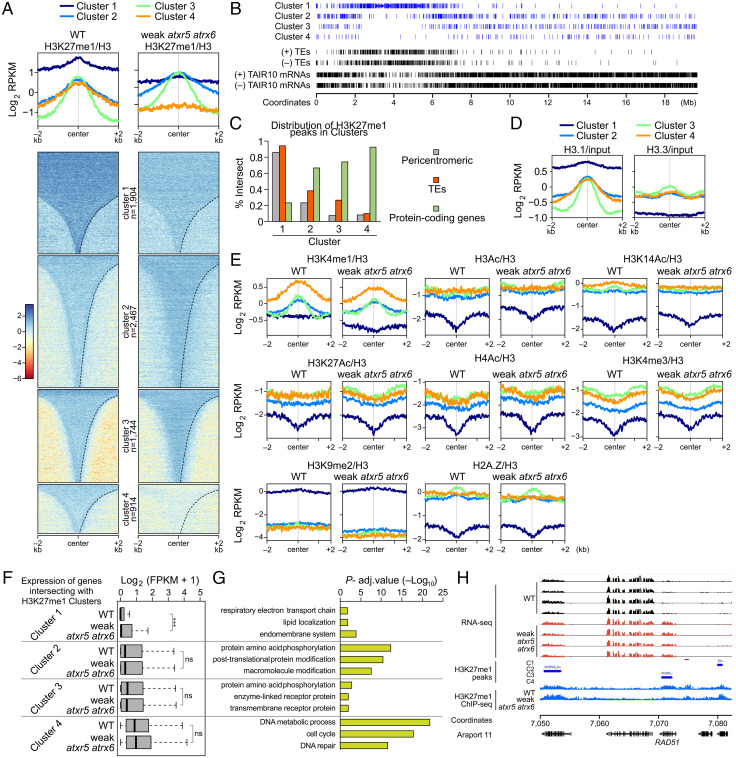
Characterization of chromatin in wild-type and *atxr5/6* (W). (*A*) Profile and heatmap of normalized (log2 RPKM) H3K27me1 signal over H3K27me1-enriched peaks in wild type (MACS2, *q* < 0.01), grouped by *k*-means clustering (cluster *n* = 4) and ordered by region length. (*B*) Distribution of enriched regions for the four H3K27me1 clusters as defined in *A* over chromosome 2. (*C*) Overlap of regions in each of the four H3K27me1 clusters from *A* with pericentromeric regions (gray), TEs (red), and protein-coding genes (green). (*D*) Distribution of normalized (RPKM) H3.1 and H3.3 ChIP-seq signal (IP over input) in wild-type over H3K27me1 clusters (*n* = 4). Data are used with permission from Stroud et al. (49). (*E*) Distribution of H3K4me1, H3Ac, H3K14Ac, H3K27Ac, H4Ac, H3K4me3, H3K9me2, and H2A.Z and H3.3 ChIP-seq signal normalized to H3 in wild-type and *atxr5/6* (W) cotyledons over the four H3K27me1 clusters. (*F*) Boxplot of RNA-seq log2 expression (average FPKM + 1) for genes overlapping each of the four H3K27me1 clusters in wild-type and *atxr5/6* (W) cotyledons. Center lines indicate the median, upper and lower bounds represent the 75th and 25th percentiles, respectively, whiskers indicate the minimum and the maximum, and outliers are not plotted. *n* = 4 independent replicates in wild-type and *atxr5/6* (W) cotyledons were averaged. Unpaired two-sample Wilcoxon test was used to test for significant differences in average expression in wild type vs. *atxr5/6* (W) in each cluster: not significant (ns), *P* > 0.05; ***P ≤ 0.001. (*G*) GO term analysis for genes overlapping regions from each of the four H3K27me1 clusters. A −log adjusted *P* value is shown for three representative GO classes for each cluster. (*H*) Normalized H3K27me1 ChIP-seq signal (reads per kilobase of transcript per million mapped reads [RPKM]) and normalized RNA-seq signal (RPKM) in wild-type and *atxr5/6* (W) cotyledons over *RAD51*. All RNA-seq tracks use same scale, as do both ChIP-seq tracks.

To characterize changes in H3K27me1 in more detail, we identified 7,029 regions normally enriched for H3K27me1/H3 in wild type, and grouped them into four clusters according to their behavior in the *atxr5/*6 (W) mutant using *k*-means clustering. Clusters 1 and 4 both showed a reduction in H3K27me1 in *atxr5/*6 (W) (Fig. 2*A*). H3K27m1 is also known to be regulated by the H3K27 demethylase REF, and indeed clusters 2 and 3 lost H3K27me1 in the *ref*-5 mutant and slightly gained H3K27me1 in the *atxr5/*6 (W) mutant, reflecting the activity of this alternative pathway in the H3K27me1 deposition (*SI Appendix*, Fig. S6) (15). Cluster 1 regions had the highest H3K27me1 levels in wild type (Fig. 2*A*), were predominantly concentrated in pericentromeric heterochromatin, and highly overlapped with TEs (Fig. 2 *B* and *C*), while clusters 2, 3, and 4 had lower wild-type H3K27me1 levels and mostly occurred over protein-coding genes in the chromosome arms (Fig. 2 *A*, *B*, and *C*). Additionally, cluster 1 contained much higher levels of H3.1, along with less H3.3, than the other three clusters (Fig. 2*D*). It has been well-characterized that ATXR5/6 have a preference for methylating H3.1 over the H3.3 histone variant (2), which suggests that the higher H3K27me1 levels in the pericentromere may be facilitated by abundant H3.1. We also examined whether any of the other chromatin marks we profiled were altered in *atxr5/6* (W) in a cluster-specific manner (Fig. 2*E*). Notably, H3K4me1 was decreased in *atxr5/6* relative to wild type in cluster 1 and to a lesser extent in cluster 4, while we observed a mild increase in several activating histone modifications in cluster 1 such as H3Ac, H3K14Ac, and H3K4me3.

In order to better understand the effect of H3K27me1 on gene expression, we plotted H3K27me1 and other histone modifications in wild type according to gene expression levels (*SI Appendix*, Fig. S7*A*). We observed that H3K27me1 was enriched over moderately to lowly expressed genes containing low-to-intermediate levels of activating histone marks. Interestingly, H3K27me1-marked genes were mostly distinct from genes marked with H3K27me3 (*SI Appendix*, Fig. S7 *B* and *C*).

We next intersected H3K27me1-enriched clusters with protein-coding genes and plotted their expression in wild type and *atxr5/6* (W) (Fig. 2*F*). Genes in cluster 1 but not clusters 2, 3, and 4 were significantly up-regulated in *atxr5/6* (W) (Fig. 2*F*). Gene Ontology (GO) term analysis revealed distinct classes of genes in each cluster (Fig. 2*G*). Strikingly, genes assigned to cluster 4 were very strongly enriched in cell cycle– and DNA repair–related GO terms, and included AtRAD51, AtBRCA1, PARP2, and AtGR1 (for example, Fig. 2*H*). Up-regulation of these and other DNA repair genes is a hallmark of the *atxr5/6* (W) phenotype (10, 12). While it is intriguing that these genes lose H3K27me1 and are up-regulated in *atxr5/6* (W), the significance of this is unclear because these DNA repair genes represent only a subset of H3K27m1-enriched genes in cluster 4, and most cluster 4 genes are not up-regulated in *atxr5/6* (*SI Appendix*, Fig. S8).

**Fig. 3.**
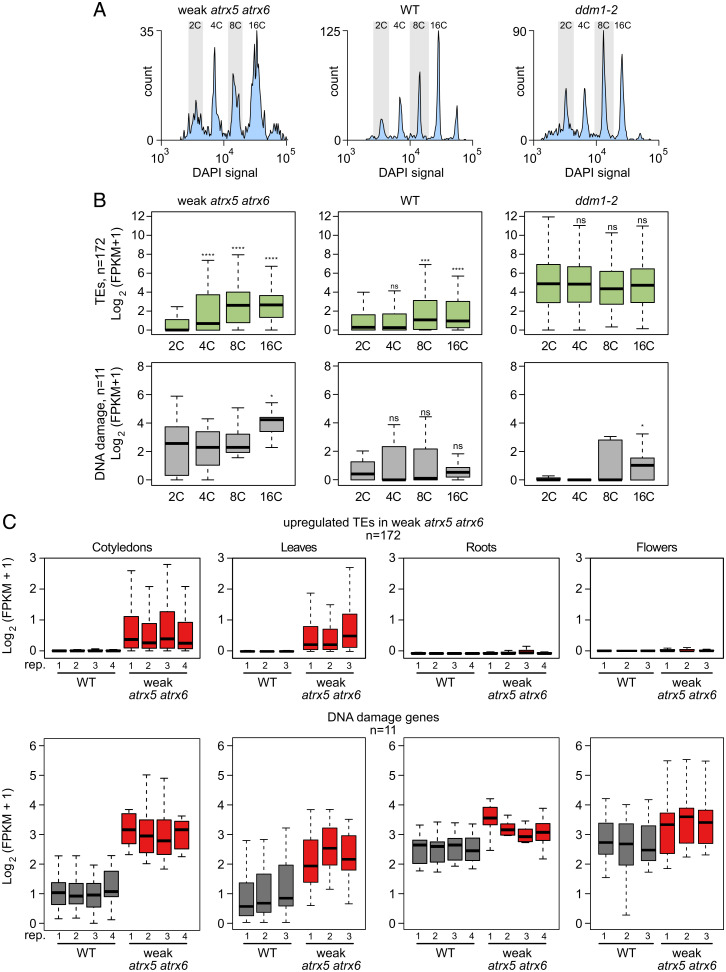
Characterization of ploidy- and tissue-specific transcriptional profiles in wild type, *atxr5/6* (W), and *ddm1-2* mutants. (*A*) Representative flow cytometry profiles of wild-type, *atxr5/6* (W), and *ddm1-2* cotyledons used to generate Smart-seq2 libraries. For Smart-seq2, each replicate consisted of a pool of 50 nuclei sorted from the indicated peak of the histogram (peaks labeled at top). (*B*) Boxplot of RNA-seq log2 expression (average FPKM + 1) of TEs (*Top*) and DNA damage genes (*Bottom*) detected in Smart-seq2 libraries as a function of ploidy in wild-type, *atxr5/6* (W), and *ddm1-2* cotyledons. Center lines indicate the median, upper and lower bounds represent the 75th and 25th percentiles, respectively, whiskers indicate the minimum and the maximum, and outliers are not plotted. Values plotted represent an average of two to four independent replicates. Unpaired two-sample Wilcoxon test was used to determine significance between 2C and 4C, 8C, and 16C nuclei for the indicated samples: ns, *P* value > 0.05; *P ≤ 0.05, **P ≤ 0.01, ***P ≤ 0.001, ****P ≤ 0.0001. (*C*) Boxplot of RNA-seq log2 expression (average FPKM + 1) of TEs (*Top*) and DNA damage genes (*Bottom*) in cotyledons, roots, leaves, and flowers in wild type and *atxr5/6* (W) mutant (three or four replicates each).

A long-standing question concerning ATXR5/6 function is the relationship between the different *atxr5/6* (W) mutant phenotypes: excess DNA, transcriptional activation of TEs and DNA damage genes, and heterochromatin decondensation. Previous work found that the excess DNA and chromatin decompaction phenotypes are specific to the higher-ploidy cells in leaf tissue (11). However, RNA-seq in *atxr5/6* (W) has only been done in tissues that include cells with a range of ploidies. To examine transcriptional changes as a function of ploidy in the *atxr5/6* (W) mutant, we performed RNA-seq of pools of 50 fluorescence-activated cell sorting (FACS)–sorted 2C, 4C, 8C, and 16C nuclei from cotyledons (Fig. 3*A*) using Smart-seq2 (19), which provides high sensitivity compared with other very low input RNA-seq methods (20). We used the well-characterized *ddm1-2* mutant as a control that shows strong TE derepression (21). After filtering out poor-quality libraries, we retained two to four replicates per ploidy/sample. Among the high-quality libraries, we detected between 10,000 and 15,000 genes with at least one read and ∼10,000 genes with at least five reads across each library, while very few genes were detected in negative control wells into which no nuclei were sorted (*SI Appendix*, Fig. S9*A* and Dataset S1). Replicates of the same ploidy tended to cluster together by both hierarchical clustering and multidimensional scaling (MDS) analysis, with 2C and 4C samples and 8C and 16C samples showing the most similarity to each other (*SI Appendix*, Fig. S9 *B* and *C*). We plotted expression levels of TEs and a selected list of DNA damage genes across all samples (Fig. 3*B*). We detected minor TE activation in 4C nuclei in *atxr5/6* (W), with a much higher activation in 8C and 16C nuclei (Fig. 3*B*). These TEs were largely silent in wild type, and were highly up-regulated regardless of ploidy in *ddm1-2* (Fig. 3*B*). These data suggest that the TE overexpression phenotype is correlated with the excess DNA and chromocenter decompaction phenotypes in *atxr5/6* (W), which also occur only at higher ploidies (Fig. 3 *A* and *B*). However, DNA damage response genes were broadly up-regulated in *atxr5/6* (W) at all ploidy levels, including in 2C nuclei (Fig. 3*B*), but were only slightly up-regulated in *ddm1-2* nuclei. DNA damage genes were also up-regulated in *atxr5/6* (W) roots and to a lower extent in young floral tissues, despite these tissues showing minimal TE derepression (Fig. 3*C*). We next profiled H3K27me1 changes in floral tissue to determine if increased expression of H3K27me1-enriched DNA damage–related genes in *atxr5/6* (W) was also accompanied by loss of H3K27me1. Though there were no differences in H3K27me1 levels in the pericentromeres as a whole in floral tissue (*SI Appendix*, Fig. S10*A*), or at TEs (*SI Appendix*, Fig. S10*B*), cluster 4 regions had decreased H3K27me1 levels in *atxr5/6* (W) in flower buds (*SI Appendix*, Fig. S10*C*). This suggests either that the reduction of *ATXR6* function in floral tissue (Fig. 1*C*) reduces H3K27me1 at cluster 4 genes, causing transcriptional activation of some genes, or alternatively that transcriptional activation of these genes may inhibit maintenance of H3K27me1 at these loci. In either case, these data show that the up-regulation of DNA damage response genes in *atxr5/6* (W) is a separable process from the chromocenter remodeling and TE activation phenotypes.

### Multiple Distinct Mechanisms for *atxr5/6* Suppression.

**Fig. 4.**
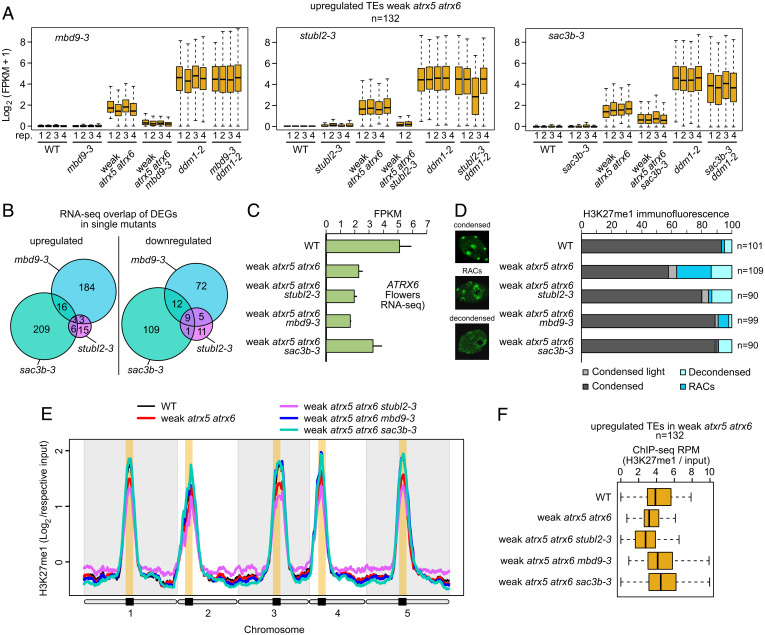
Distinct mechanisms of *atxr5/6* (W) suppression. (*A*) Boxplot of average expression of TEs up-regulated in *atxr5/6* (W) cotyledons for the indicated mutants and replicates. Center lines indicate the median, upper and lower bounds represent the 75th and 25th percentiles, respectively, whiskers indicate the minimum and the maximum, and outliers are not plotted. (*B*) Overlap between significantly (log2 fold change ≥ 1, false discovery rate ≤ 0.05) up-regulated and down-regulated genes for each mutant vs. wild type in cotyledons. (*C*) Bar chart of RNA-seq FPKM values for *ATXR6* expression in wild-type, *atxr5/6* (W), *stubl2-3 atxr5/6* (W), *mbd9-3 atxr5/6* (W), and *sac3b-3 atxr5/6* (W) flowers. Bars represent mean and whiskers represent ±SE (SEM) from *n* = 3 biological replicates. (*D*) Immunofluorescence and quantification of chromocenter appearance in H3K27me1-stained leaf nuclei from wild type, *atxr5/6* (W), *stubl2-3 atxr5/6* (W), *mbd9-3 atxr5/6* (W), and *sac3b-3 atxr5/6* (W). (*E*) Genome-wide H3K27me1 ChIP-seq signal normalized by input for the indicated samples in leaves. Smoothed log2 ratio of normalized (RPM) ChIP-seq signal over 100-kb windows is shown. (*F*) Boxplot of log2(H3K27me1/input) ChIP-seq signal over TEs up-regulated in the *atxr5/6* (W) mutant in leaves of the indicated genotypes.

We previously performed a forward genetic screen for suppressors of the *atxr5/6* (W) mutant phenotypes, which identified mutations in genes encoding a methyl-CpG–binding domain protein (MBD9), two components of the TREX-2 complex, SAC3B and THP1, and a SUMO-interacting E3 ubiquitin ligase (STUbL2) (12). These mutations suppressed the TE activation phenotype of *atxr5/6* (W) (12). To investigate if these mutations also suppress TE activation in other mutant backgrounds, we crossed *mbd9-3*, *sac3b-3*, and *stubl2-3* with *ddm1-2*, a mutant that displays strong TE derepression in pericentromeric heterochromatin (10). RNA-seq in cotyledons revealed that neither *mbd9-3* nor *stubl2-3* suppressed TE up-regulation in the *ddm1-2* background (Fig. 4*A*). *sac3b-3* moderately suppressed TE up-regulation in *ddm1-2* (Fig. 4*A*), but this effect was limited to a small subset of 98 highly up-regulated TEs (*SI Appendix*, Fig. S11). Thus, the suppression of TE activation by these mutants was mostly specific to the *atxr5/6* (W) mutant background.

To learn more about the pathways affected by the suppressor mutations, we generated complementing epitope-tagged transgenic lines for MBD9 and SAC3B (*SI Appendix*, Fig. S12). We were unable to generate complementing transgenic lines for STUbL2, presumably because the epitope tags interfered with protein function. We performed IP mass spectrometry of epitope-tagged MBD9 and SAC3B in their respective single-mutant and *atxr5/6* (W) triple-mutant backgrounds. As previously reported, MBD9 interacted with the SWR1 complex and CHR11/17 remodelers (22), and these interactions persisted in the *atxr5/6* (W) background (Table 3). Furthermore, when we crossed two highly conserved components of the SWR1 complex, *arp6-1* and *sef1-1*, to *atxr5/6* (W), we observed a suppression of the *atxr5/6* (W) DNA overreplication and TE up-regulation phenotypes (*SI Appendix*, Fig. S13). This suggests that *mbd9-3* suppresses *atxr5/6* (W) phenotypes via its participation in the SWR1 complex. For SAC3B, we observed interaction with THP1 and several nucleoporin-related proteins (Table 3). Importantly, MBD9- and SAC3B-interacting proteins were largely nonoverlapping, and neither MBD9 nor SAC3B pulled down STUbL2 (Table 3 and Dataset S2). These data suggest that MBD9, SAC3B, and STUbL2 may act in different pathways and suppress the *atxr5/6* (W) phenotype via distant mechanisms. In further support of this, we performed RNA-seq of *mbd9-3*, *sac3b-3*, and *stubl2-3* cotyledons and found that the overlaps between the misregulated genes in each of these mutants were relatively low, although they were significant when measured by a hypergeometric test (*P* ≤ 0.0001) (Fig. 4*B*). Furthermore, we plotted the expression of the differentially expressed genes (DEGs) identified for each mutant across all the genotypes and generally found little coregulation of these genes in the different mutants (*SI Appendix*, Fig. S14).

**Table 3.** IP mass spectrometry: spectral counts for MBD9-9×Myc, MBD9-3×FLAG, and SAC3B-3×FLAG lines representing enriched proteins over wild type

Protein	Description	MBD9-9× MYC *mbd9- 3_*R1	MBD9- 3×FLAG *mbd9- 3_*R1	MBD9- 3×FLAG *weak atxr5 atxr6 mbd9- 3_*R1	MBD9- 3×FLAG *weak atxr5 atxr6 mbd9- 3_*R2	MBD9- 3×FLAG *weak atxr5 atxr6 mbd9- 3_*R3	SAC3B- 3×FLAG * sac3b- 3_*R1	SAC3B- 3×FLAG *weak atxr5 atxr6 sac3b- 3_*R1	SAC3B- 3×FLAG *weak atxr5 atxr6 sac3b- 3_*R2	SAC3B- 3×FLAG *weak atxr5 atxr6 sac3b- 3_*R3	WT-3× FLAG_R1	WT-3x FLAG_R2	WT-9× MYC_R1	*weak atxr5 atxr6*-3× FLAG_R1	*weak atxr5 atxr6*-3× FLAG_R2
AT3G01460	MBD9	355	607	571	218	177	3	0	0	0	0	0	0	0	0
AT2G17930	TRA1A	128	76	91	47	27	2	0	3	2	0	0	0	12	0
AT4G36080	TRA1B	120	74	85	38	24	0	0	2	2	0	0	0	11	0
AT3G12810	PIE1	75	49	30	7	14	3	0	0	0	0	0	0	0	0
AT2G47210	SWC4	12	9	6	3	5	5	0	0	0	0	0	0	3	0
AT5G45600	YAF9A	5	6	3	0	0	0	0	0	0	0	0	0	0	0
AT3G33520	ARP6	16	5	9	0	4	2	0	0	0	0	0	0	0	0
AT2G36740	SWC2	17	0	2	0	2	0	0	0	0	0	0	0	0	0
AT2G02470	AL6	2	0	0	0	0	0	0	0	0	0	0	0	0	0
AT5G37055	SEF	2	0	0	0	0	0	0	0	0	0	0	0	0	0
AT5G18620	CHR17	122	190	166	94	70	6	4	2	2	3	0	0	3	2
AT3G06400	CHR11	118	177	177	94	61	5	5	2	3	4	0	0	3	3
AT3G06290	SAC3B	0	0	0	0	4	391	245	104	104	38	0	0	0	0
AT2G19560	THP1	0	0	0	0	0	42	28	17	17	0	0	0	0	0
AT4G11790	Plekstrin homology	0	0	0	0	0	21	21	10	9	0	0	0	0	0
AT5G20200	Nucleoporin- related	0	0	0	0	0	21	12	4	3	0	0	0	0	0
AT1G67180	STUbL2	0	0	0	0	0	0	0	0	0	0	0	0	0	0

We have shown that higher levels of tissue-specific residual ATXR6 correlate with the severity of the *atxr5/6* (W) phenotype (Fig. 1), suggesting that increasing *ATXR6* transcript levels could be a mechanism for rescue by other factors. We therefore tested whether the suppression of the *atxr5/6* (W) phenotype by *mbd9-3*, *sac3b-3*, and *stubl2-3* was a result of up-regulation of *ATXR6*. We performed RNA-seq in flowers, where ATXR6 expression in *atxr5/6* (W) is relatively high, so that changes in ATXR6 expression would be easier to detect. ATXR6 expression increased in the *sac3b-3 atxr5/6* (W) background as compared with *atxr5/6* (W) (Fig. 4*C*), suggesting that *sac3b-3* may indeed be rescuing *atxr5/6* (W) by up-regulating *ATXR6* transcription. However, we did not detect an increase in *ATXR6* expression in either *mbd9-3 atxr5/6* (W) or *stubl2-3 atxr5/6* (W) (Fig. 4*C*). These observations were confirmed by qRT-PCR in seedling tissue (*SI Appendix*, Fig. S15). To further test the hypothesis that up-regulation of ATXR6 expression may be sufficient to suppress the *atxr5/6* phenotype, we crossed *atxr5/6* (W) with *mediator12* (*med12*) (23), which has pleiotropic effects on gene expression. Loss of *MEDIATOR12* increased ATXR6 expression and suppressed the TE up-regulation and extra DNA phenotypes of *atxr5/6* (W) (*SI Appendix*, Fig. S16). These results further confirm that up-regulation of ATXR6 transcript can suppress the *atxr5/6* (W) phenotype.

We further explored the mechanisms by which *mbd9-3* and *stubl2-3* rescue the *atxr5/6* (W) phenotype. To determine if the suppression of *atxr5/6* (W) involves the compaction of the chromocenters, we performed immunofluorescence of H3K27me1 and observed that all mutants suppressed the chromocenter decompaction phenotype of *atxr5/6* (W) (Fig. 4*D*). Additionally, we found by ChIP-seq that H3K27me1 levels in *atxr5/6* (W) were restored to wild-type levels in chromocenters by both *mbd9-3* and *sac3b-3* (Fig. 4 *E* and *F* and *SI Appendix*, Fig. S17). In *sac3b-3* this likely reflects increased ATXR6 expression (Fig. 4*C*). However, loss of MBD9 also partially restored H3K27me1 levels in *atxr5/6* (W), suggesting that MBD9 may affect H3K27me1 deposition via an unknown mechanism. Interestingly, *stubl2b-3* suppressed the *atxr5/6* (W) phenotype without increasing H3K27me1 levels (Fig. 4 *E* and *F* and *SI Appendix*, Fig. S17), suggesting that *STUBL2* acts downstream of H3K27me1 or possibly via an independent mechanism. H3K27me1 levels were also unaffected in the single *stubl2-3* mutant (*SI Appendix*, Fig. S18), suggesting that *stubl2-3* is not involved in the regulation of H3K27me1 levels. We also checked whether *sac3b-3*, *mbd9-3*, and *stubl2-3* result in transcriptional misregulation of other factors that may indirectly result in suppression of the *atxr5/6* (W) phenotypes, for instance by affecting histone H3.1/H3.3 deposition. However, we did not find evidence of this in our RNA-seq datasets (Dataset S3). Together, these data suggest that all three mutants may rescue *atxr5/6* (W) via distinct mechanisms.

## Discussion

Understanding how chromatin influences genomic stability has important implications for cellular and organismal viability. In *Arabidopsis*, the heterochromatin-specific mark H3K27me1 has emerged as an important connection between epigenetic gene silencing and genome stability. Reduction of H3K27me1 in the *atxr5/6* (W) mutant results in up-regulation of TEs, activation of DNA damage response genes, chromocenter decompaction, and a genomic instability defect characterized by accumulation of heterochromatin-derived DNA in certain endoreduplicated tissues. The goal of this study was to investigate the function of the H3K27me1 epigenetic mark by specifically addressing several questions: 1) What contributes to the tissue-specific defects observed in the *atxr5/6* mutants? 2) How does the epigenetic landscape change upon the reduction of H3K27me1? 3) What is the connection between the phenotypes resulting from a reduction in H3K27me1? 4) How might characterization of the *atxr5/6* (W) suppressors identified previously in our screen inform us about the biology of the ATXR5/6 methyltransferases?

Through an examination of gene expression across tissue types and its correlation with changes in H3K27me1 levels, we made several interesting observations. A key finding from this work is that the *atxr6* allele used here and in prior studies is a weak allele that still produces low but functional levels of *ATXR6* transcript in some tissues. We observed that the tissue-specific defects in *atxr5/6* (W) mutants likely reflect varying levels of residual expression of *ATXR6* in these tissues. Despite both roots and cotyledons displaying similar levels of endoreduplication, *ATXR6* was much more highly expressed in roots, while the *atxr5/6* (W) phenotype is present in cotyledons but absent from roots. These observations suggest that there is a minimum amount of ATXR6 required to maintain H3K27me1 levels and, if *ATXR6* expression levels are reduced below this threshold, a critical amount of H3K27me1 is lost and the *atxr5/6* (W) phenotypes occur. In support of this, complete loss of both *ATXR5* and *ATXR6* results in female gametophytic lethality, suggesting a need for a minimal amount of H3K27me1 to be maintained for viability. This model is also supported by the observation that in flowers, where *ATXR6* transcript levels are only reduced ∼50% in the weak mutant, H3K27me1 levels at chromocenters were unaffected. Furthermore, we identified two suppressors, *sac3b-2* and *med12*, which restored H3K27me1 levels and suppressed *atxr5/6* (W) phenotypes, likely by increasing *ATXR6* RNA expression.

Unfortunately, we were not able to detect *ATXR6* expression across the endoreduplicated nuclei to determine if the TE derepression that increased with the levels of endoreduplication in the *atxr5/6* (W) mutant correlates with a decrease in *ATXR6* levels, as our model would predict. Alternatively, it is possible that the increase in TE derepression occurred as a result of an increase in copy number upon endoreduplication. However, we would then predict that the same TEs would increase in the *ddm1-2* mutant but instead they remained constant. We thus attribute the increase in TE derepression to increased chromocenter defects that also increase with ploidy in the *atxr5/6* (W) mutant, rather than to endoreduplication itself, but we cannot exclude its involvement. Several studies suggest that the relationship between gene expression and genome duplication might be complex and that gene expression does not strictly correlate with genome doubling in *Arabidopsis* (24, 25). Further studies are required to address how gene expression dosage is regulated upon endoreduplication.

One phenotype that did not strictly correlate with *ATXR6* expression was the up-regulation of DNA damage genes. This occurred in *atxr5/6* (W) regardless of *ATXR6* levels in a given tissue. This suggests that the DNA damage response may be a separate process from the TE derepression, chromocenter decompaction, and excess DNA production seen in the *axr5/6* (*W*) mutant, and that it may be more sensitive to the partial loss of *ATXR6*. In particular, it seems possible that some form of genome instability or replication stress occurs in *atxr5/6* (W), causing induction of DNA damage response genes. Indeed, similar sets of DNA damage genes are also activated in mutants of genes involved in DNA replication, such as in *FAS1* and *FAS2*, both components of the chromatin assembly factor 1 (CAF-1) chaperone complex, as well as in *BRU1*, *RFC1*, and mutations in DNA polymerases, *Pol alpha* (α), *delta* (δ), and *epsilon* (ε) (17, 26–31).

We profiled various histone modifications in our study to determine which other chromatin changes may be associated with the molecular phenotypes observed in the *atxr5/6* (W) mutant. Although we observed a few minor changes, including increased activating histone modifications at TEs up-regulated in *atxr5/6* (W) and a slight increase in H3K9me2 and decrease in H3K4me1 over pericentromeric heterochromatin, we found no major changes in histone modifications other than loss of H3K27me1 in *atxr5/6* (W). This suggests that H3K27me1 reduction may be the major epigenetic change responsible for the molecular phenotypes observed in the *atxr5/6* (W) mutant. We defined a subset of protein-coding genes that strongly lost H3K27me1 in the *atxr5/6* (W) mutant and, interestingly, this subset included DNA damage genes that are up-regulated in *atxr5/6* (W). Additional studies are needed to determine what function, if any, H3K27me1 plays in regulating the expression of these genes, or if the losses of H3K27me1 at these genes are a secondary consequence of their up-regulation. We also identified a subset of H3K27me1-enriched regions that slightly gained H3K27me1 in the *atxr5/6* (W) mutant, and these sites were enriched for REF H3K27me2/3 demethylase activity, highlighting the multiple mechanisms of H3K27me1 regulation.

We also explored the mechanisms by which mutation of three genes was previously shown to suppress *atxr5/*6 (W) TE up-regulation phenotypes. We found that both *sac3b-3* and *mbd9-3* suppressed *atxr5/6* (W) by restoring H3K27me1 levels at chromocenters. For *sac3b-3*, the mechanism likely involves increasing *ATXR6* expression in the hypomorphic *atxr6* (W) allele. How *mbd9-3* rescues H3K27me1 levels remains unclear, but one possibility is that it could act via regulation of other genes involved in H3K27me1 maintenance. Interestingly, the third mutant, *stubl2-3*, suppressed the *atxr5/6* (W) TE phenotype without restoring H3K27me1 levels, suggesting it functions downstream of H3K27me1 in regulating TE expression. As previously described, STUbL2 is the only suppressor identified from the previous screen that is coexpressed during G1/S phase with ATXR6 and other factors involved in DNA replication. This suggests that STUbL2 may be more directly involved in the initial events that occur upon a reduction in H3K27me1 levels. Further work is needed to understand the mechanism of action of STUbL2.

In summary, this work provides an examination of tissue-specific transcriptional and chromatin changes occurring in the *atxr5/6* (W) mutant, and provides important considerations for future work. Most notably, our study revealed that due to the hypomorphic nature of the *atxr5/6* (W) mutant, future work involving suppressors or enhancers of the *atxr5/6* (W) mutant requires systematic profiling of their effect on *ATXR6* and H3K27me1 levels in order to assess their direct or indirect impact on H3K27me1 biology.

## Materials and Methods

### Plant Materials.

All *Arabidopsis* plants used in this study were of the Col-0 ecotype and were grown at 22 °C under long-day conditions (16 h light, 8 h dark). The following *Arabidopsis* mutant lines were used: *mbd9-3* (SALK_039302), *arp6-1* (SAIL_599_G03), *sef-1* (SAIL_536_A05), *stubl2-3* (GABI_910B12), *atxr5-1* (SALK_130607), *atxr6-1* (SAIL_240_H01), *atxr6-2* (SALK_206371), *ddm1-2^21^*, *med12* (SALK_108241c), and *sac3b-3* (SALK_065672).

### qRT-PCR.

Total RNA was prepared from 2-wk-old shoots (∼3 shoots per replicate) and 2-wk-old roots (∼5 roots per replicate) using the Direct-zol RNA MiniPrep Kit (R2050; Zymo Research). RNA (1 to 2 µg) was used for the preparation of complementary DNA using SuperScript III First-Strand Synthesis SuperMix (18080-400; Invitrogen). Signal detection, quantification, and normalization were done using CFX Maestro software (Bio-Rad).

### RNA-Seq and Analysis.

Total RNA was extracted from 2-wk-old cotyledons (∼20 cotyledons per replicate), 2-wk-old roots (∼5 roots per replicate), 4-wk-old flowers (∼4 flower buds from separate plants per replicate), and 4-wk-old rosette leaves (1 leaf from separate plants per replicate) grown on 1% Murashige and Skoog medium or soil under long-day conditions. RNA was extracted using the Direct-zol RNA Miniprep Kit. For RNA-seq, 1 µg of total RNA was used to prepare libraries using the Illumina TruSeq Stranded mRNA-Seq Kit or the Illumina NeoPrep Kit. Libraries were sequenced on either an Illumina HiSeq 4000 or NovaSeq 6000 instrument. Reads were aligned to TAIR10 using TopHat (32), allowing up to two mismatches and only keeping reads mapped to one unique location. FPKM (fragments per kilobase of transcript per million fragments mapped) values and differential gene expression were analyzed using Cufflinks (32) with default settings. GO term enrichment was determined using agriGO (33).

### Smart-seq2.

Fresh nuclei from 2-wk-old cotyledons were extracted as follows. Roughly 50 cotyledons were finely chopped with a razor into 50 μL Partec nucleus extraction buffer (Sysmex America; 05-5002) and DAPI-stained with 400 μL Partec nucleus staining buffer. Samples were filtered once through a 35-μm nylon mesh (Falcon; 352235) and subjected to fluorescence-activated nucleus sorting. Nuclei were sorted from the 2C, 4C, 8C, and 16C peaks based on DAPI fluorescence. Fifty nuclei from each peak were sorted into individual wells of a 96-well plate. Negative controls, with no nuclei sorted into a well, were also included in every plate and carried through library preparation and sequencing. Libraries were prepared according to the Smart-seq V2 protocol at reduced volume and with a few modifications as described previously (34), and sequenced on an Illumina NovaSeq 6000. Reads were aligned to TAIR10 with chloroplasts and mitochondria excluded using HISAT2 (35) with default settings. SAMtools (36) view was used to filter uniquely mapped proper pairs with the following parameters: -b -q 60 -f 2. Duplicate reads were removed using Picard tools MarkDuplicates.jar (https://broadinstitute.github.io/picard/). Counts for each gene were generated using HTSeq-count (37) using Araport11 (38) gene and TE annotations. FPKM values and differential gene expression were analyzed using Cuffdiff (32) with default settings. The R cummeRbund package (39) was used to generate a dendrogram of Jensen–Shannon distance between sample replicates and the MDS plot for sample replicates for all genes.

### Epitope-Tagged Transgenic SAC3B and MBD9 Lines.

Full-length genomic DNA fragments containing ∼1 kb (MBD9) or ∼1 kb (SAC3B) of promoter sequence, together with genomic gene sequences up to the annotated/major stop codon of MBD9 and SAC3B, were amplified using PCR (*SI Appendix*). The PCR product was cloned into the pENTR/D vector (Invitrogen) and delivered into a modified pEG destination vector containing 3×FLAG and 9×MYC tags using the LR Reaction Kit (Invitrogen). The pEG destination vectors were transformed into *Agrobacterium* strain AGL0, and transformed into plants using the floral dip method (40).

### Affinity Purification and Mass Spectrometry.

Approximately 10 g of flowers from transgenic lines expressing SAC3B-3×FLAG, MBD9-3×FLAG, or MBD9-9×MYC, and from Col-0 and *atxr5/6* (W) plants as negative controls, was ground to a fine powder using a RETCH homogenizer (3 min at 30 Hz/min) and suspended in 30 mL of IP buffer. Tissue was further dounce-homogenized until lump-free, and then centrifuged for 20 min at 4,000 × *g* and 4 °C. The lysate was filtered through two layers of Miracloth. Supernatant was incubated with 200 μL anti-FLAG M2 magnetic beads (M8823; Sigma) at 4 °C for 2 h. The bead-bound complex was washed once with 10 mL IP buffer and then four times (5 min rotating at 4 °C with 1.5 mL IP buffer), followed by a final wash with IP buffer without Nonidet P-40. The FLAG IP was eluted twice with 300 µL 250 µg/mL 3× FLAG peptides (Sigma; F4799) in TBS (50 mM Tris⋅Cl, pH 7.5, 150 mM NaCl), mixing for 15 min at 4 °C. The eluted protein complexes were precipitated by trichloroacetic acid (TCA) and subjected to mass spectrometry analyses as previously described (41). In the case of MBD9-9×Myc, monoclonal 9E10 coupled to magnetic beads was used (88842; Pierce). The bead-bound complexes were washed six times with 1 mL of IP buffer. For each wash, the beads were rotated at 4 °C for 5 min. For MBD9-9×Myc, proteins were eluted twice with 100 µL 8 M urea in 50 mM Tris (pH 8.5), mixing for 15 min at 37 °C. The supernatant was TCA-precipitated.

### Flow Cytometry and FACS.

All flow cytometry analysis and FACS were performed as previously described (12). Typically, cotyledons from 20 plants were pooled, for root tissue ∼10 roots were pooled, and for leaf tissue 3 leaves were pooled. DNA-seq libraries were generated as previously described (12). Briefly, DNA from 10,000 FACS-sorted 16C nuclei was isolated using a PicoPure DNA Extraction Kit (KIT0103; Arcturus), and libraries were generated using the NuGEN Ovation Ultralow System V2 Kit according to the manufacturer’s instructions.

### Immunofluorescence.

Immunofluorescence was performed as described previously using the yH3K27me1 (Millipore; 07448) antibody (1). Representative images were taken at the Broad Stem Cell Research Center Microscopy Core at the University of California, Los Angeles (UCLA) on a Zeiss Elyra superresolution SIM/PALM microscope. *z* series images of individual nuclei were processed using Structured Illumination imaging software. Decompaction of chromocenters was scored on a Zeiss Axio Imager.D2 fluorescence microscope.

### Genome-Wide ChIP-Seq and Library Generation.

For low-input ChIP, roughly three tubes (1.5 mL) of 2-wk-old cotyledons were collected and frozen at −80 °C. Tissue was grounded to fine powder by mortar and pestle followed by in vitro cross-linking for 10 min at room temperature with 12.5 mL of nuclear isolation buffer containing 1% formaldehyde (50 mM Hepes, 1 M sucrose, 5 mM KCl, 5 mM MgCl_2_, 0.6% Triton X-100, 0.4 mM phenylmethanesulfonylfluoride [PMSF], 5 mM benzamidine, and 1× protease inhibitor mixture tablet [Roche; 14696200]). Cross-linking was stopped with 850 μL 2 M glycine rotating for 10 min at room temperature. Lysate was filtered through one layer of Miracloth and centrifuged for 20 min at 2,880 × *g* and 4 °C. The pellet was resuspended with 1 mL extraction buffer 2 (0.25 M sucrose, 10 mM Tris⋅HCl, pH 8, 10 mM MgCl_2_, 1% Triton X-100, 5 mM β-mercaptoethanol [BME], 0.1 mM PMSF, 5 mM benzamidine, and 1× protease inhibitor mixture tablet), followed by centrifugation for 10 min at 12,000 × *g* and 4 °C. The pellet was lysed with 600 µL nucleus lysis buffer on ice (50 mM Tris, pH 8, 10 mM ethylenediaminetetraacetate [EDTA], 1% sodium dodecyl sulfate [SDS], 0.1 mM PMSF, 5 mM benzamidine, and 1× protease inhibitor mixture tablet). The sample was split into two 15-mL Falcon tubes (300 μL each) and 1.275 mL of ChIP dilution buffer (1.1% Triton X-100, 1.2 mM EDTA, 16.7 mM Tris, pH 8, 167 mM NaCl, 0.1 mM PMSF, 5 mM benzamidine, and 1× protease inhibitor mixture tablet) was added and DNA was sheared on a Bioruptor Plus (Diagenode) (30 s on/30 s off, maximum power, 17 min at 4 °C). Sheared chromatin was centrifuged twice at maximum speed for 10 min at 4 °C. The supernatant was combined and further diluted with ChIP dilution buffer up to 6 mL; 100 µL of sample was saved as input and the rest was divided into seven IPs containing ∼850 μL of chromatin in 1.5-mL DNA LoBind tubes (Eppendorf; 022431021). The following antibodies were used for IPs: 5 μL of yH3 (Abcam; 1791), 5 μL of yH3K4me3 (Millipore; 04-745), 5 μL of yH3Ac (Active Motif; 39140), 5 μL of yH4Ac (Active Motif; 39244), 3 μL of yH2A.Z [polyclonal antibodies specific to *Arabidopsis* H2A.Zs HTA11 and HTA9 (22)], 5 μL of yH3K9me2 (Abcam; 1220), 10 μL of yH3K27Ac (Abcam; 4729), 15 μL of yH4K14Ac (Abcam; 52946), 20 μL of yH3K4me1 (Abcam; 889550), 10 μL of yH3K27me1 (Millipore; 07-488), and 10 μL of yH3K27me3 (Millipore; 07-449). After incubation overnight with rotation at 4 °C, 50 µL Dynabeads (equal mix of proteins A and G; Invitrogen; 10004D/10002D) was added to the chromatin and incubated for an additional 2 h. The magnetic beads were washed with 1 mL of the following buffers for 5 min rotating at 4 °C: 2× with low-salt buffer (150 mM NaCl, 0.2% SDS, 0.5% Triton X-100, 2 mM EDTA, 20 mM Tris, pH 8), 1× high-salt buffer (200 mM NaCl, 0.2% SDS, 0.5% Triton X-100, 2 mM EDTA, 20 mM Tris, pH 8), 1× LiCl wash buffer (250 mM LiCl, 1% Igepal, 1% sodium deoxycholate, 1 mM EDTA, 10 mM Tris, pH 8), and 1× with TE buffer (10 mM Tris, pH 8, 1 mM EDTA). The immunocomplex was eluted from the beads twice with 250 µL elution buffer (1% SDS, 10 mM EDTA, 0.1 M NaHCO_3_), incubating for 20 min with shaking at 65 °C. Elution buffer (400 µL) was added to the input samples. A total of 20 µL 5 M NaCl was added to each tube, and the cross-link was reversed by incubating at 65 °C overnight. Residual protein was degraded with 20 µg proteinase K in 10 mM EDTA and 40 mM Tris (pH 8) at 45 °C for 1 h followed by phenol/chloroform/isoamyl alcohol extraction and ethanol precipitation. The pellet was washed with 70% EtOH and resuspended in 50 µL molecular-grade water. Libraries were prepared using the NuGEN Ovation Ultralow System V2 Kit, according to the manufacturer’s instructions, and were sequenced on an Illumina HiSeq 4000.

For ChIP-seq in floral and leaf tissues, the ChIP was performed as previously described (42) using 5 μL yH3K27me1 (Millipore; 07-488). For ChIP-seq in leaves, 0.5 g (Fig. 4) and 2 g (*SI Appendix*, Figs. S17 and S18) of 4-wk-old rosette leaves were used per IP, and for flowers, 1 g of 4-wk-old floral buds was used per IP. Libraries were generated with a NuGEN Ovation Ultralow System V2 Kit, according to the manufacturer’s instructions, and were sequenced on either an Illumina HiSeq 4000 instrument or NovaSeq 6000.

### ChIP-Seq Analysis.

ChIP-seq fastq reads were aligned to the TAIR10 reference genome with Bowtie (43) using default settings and allowing only uniquely mapping reads. Duplicated reads were removed using SAMtools (36). The Integrated Genome Browser was used to visualize the data and to generate snapshots (44). Normalized read coverage tracks were generated using the USeq package Sam2Useq application (45) and deepTools (46). Genome-wide log2 ratio plots were generated with R (47) using normalized signal (reads per million mapped reads [RPM]) over 100-kb binned windows generated with the biotoolbox application get_datasets.pl (https://github.com/tjparnell/biotoolbox). The plots were smoothed in R (47) using the caTools library (https://cran.r-project.org/web/packages/caTools/index.html) with a moving window of 20. ChIP-seq peaks in wild type and mutants were called by the callpeak function in MACS2 (v2.1.1.) (48). ChIP-seq data metaplots and *k*-means clustering were generated by deepTools (46).

## Supplementary Material

Supplementary File

Supplementary File

Supplementary File

Supplementary File

Supplementary File

## Data Availability

All high-throughput sequencing data generated in this study are accessible at the National Center for Biotechnology Information Gene Expression Omnibus via series accession no. GSE166897 (https://www.ncbi.nlm.nih.gov/geo/query/acc.cgi?acc=GSE166897) and are listed in Dataset S4. A list of publicly available data used in this paper and a list of primers used are provided in *SI Appendix*. All study data are included in the article and/or supporting information. Previously published data were used for this work. [List of publicly available data used in this paper: from Stroud *et al.* (49), used in Fig. 2*D* and *SI Appendix*, Fig. S7: H3.3 ChIP-seq (accession no. GSM856054), H3.1ChIP-seq (accession no. GSM856055); from Antunez-Sanchez *et al.* (15), used in *SI Appendix*, Fig. S6: H3K27me1 ChIP-seq WT (sample accession no. SAMEA7583150), H3K27me1 ChIP-seq atxr5/6 (sample accession no. SAMEA7583154), H3K27me1 ChIP-seq ref-5 (sample accession no. SAMEA7583152), REF_ChIPSeq_Col (accession nos. rep 1, GSM3040333; rep 2, GSM3567218), ref_REF_ChIP-seq (accession nos. rep 1, GSM3040335; rep 2, GSM3567221; rep 3, GSM3567223).]
