# Supplementary material for: The role of ATXR6 expression in modulating genome stability and transposable element repression in Arabidopsis
Source: Proc Natl Acad Sci U S A. 2022 Jan 13;119(3):e2115570119. doi: 10.1073/pnas.2115570119 (PMC8784105; doi:10.1073/pnas.2115570119)
Supplement: Supplementary File [file pnas.2115570119.sapp.pdf]

PNAS
www.pnas.org

Supplementary Information for

The role of ATXR6 expression in modulating genome stability and transposable element repression in Arabidopsis.

Magdalena E. Potok[a,1], Zhenhui Zhong[a], Colette L. Picard[a], Qikun Liu[a,b], Truman Do[a], Cassidy E. Jacobsen[a], Ocean Sakr[a], Bilguudei Naranbaatar[a], Ruwan Thilakaratne[a], Zhanna Khnkoyan[a], Megan Purl[a], Harrison Cheng[a], Helena Vervaet[a], Suhua Feng[a], Shima Rayatpisheh[c,2], James A. Wohlschlegel[c], Ronan C. O'Malley[d,e,3], Joseph R. Ecker[d,e], and Steven E. Jacobsen[a,f,4]

Correspondence to: jacobsen@ucla.edu (S.E.J.)
Email: jacobsen@ucla.edu

This PDF file includes:

Supplementary information
Figures S1 to S18
Legends for Datasets S1 to S4
SI References

Supplementary information:

List of publicly available data used in the paper:

H3.3 – 10 day seedlings – (accession number GSM856054) [1]
H3.1 – 10 day seedlings – (accession number GSM856055) [1]
H3K27me1 WT – (sample accession number SAMEA7583150) [2]
H3K27me1 *atxr5/6* - (sample accession number SAMEA7583154) [2]
H3K27me1 *ref6-5* – (sample accession number SAMEA7583152) [2]
REF6_ChIPSeq_Col - (accession numbers, rep 1- GSM3040333, rep2 - GSM3567218) [2]
*ref6*_REF6_ChIPseq – (accession numbers, rep1-GSM3040335, rep2 - GSM3567221, rep3 - GSM3567223) [2]

List of primers used in the study:

Primer Name	Sequence	Description
JP12457	CACCGTACATCACATGTTACATGCA	Forward primer ~1kb upstream of MBD9, with CACC tag added for Gateway Cloning into P-ENTR D TOPO (Life Technologies)
JP12458	GGATCCCTCGGGTTCTTTCCT	Reverse primer -stop codon for MBD9
JP10874	CACCCAAGGGAGATGGTGACAAC	Forward primer ~1,5kb upstream of SAC3B, with CACC tag added for Gateway Cloning into P-ENTR D TOPO (Life Technologies)
JP10875	GAAGTAAATACAGAGCTTCTCGG	Reverse primer -stop codon for SAC3B
JP6292	GGAGGCATGCAGGTTCTATC	ATXR6_F_RT-qPCR
JP6293	TTCCTCCCTTCTGGTGAGTG	ATXR6_R_RT-qPCR
JP2452	TCGTGGTGGTGAGTTTGTTAC	ACTIN 7_F_RT-qPCR
JP2453	CAGCATCATCACAAGCATCC	ACTIN 7_R_RT-qPCR
JP3395	AATGTAAGTTGTAAACCATTTGAACGTGACC	SDC_F_RT-qPCR
JP3396	CAGGCATCCGTAGAACTCATGAGC	SDC_R_RT-qPCR
JP9055	AGTCCTTTTGGTTGCTGAACA	ATCOPIA28_F_RT-qPCR
JP9056	CCGGATGTAGCAACATTCACT	ATCOPIA28_R_RT-qPCR
JP9640	AACTAACGTCATTACATACACATCTTG	soloLTR_F_RT-qPCR
JP9641	AATTAGGATCTTGTTTGCCAGCTA	soloLTR_R_RT-qPCR
15076	GGACCATAGATTCTTCATTCCCTTCA	AT1TE45375_F_RT-qPCR
15077	AGATCTACTGACTTTGTAGAATCTAGGG	AT1TE45375_R_RT-qPCR

Supplementary Figures

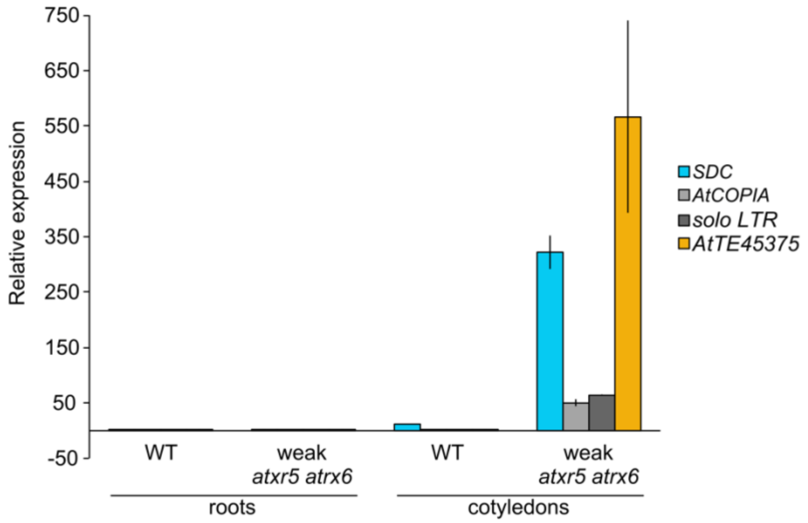

Fig. S1. RT-qPCR of four TEs or TE related genes upregulated in atxr5/6 (W) cotyledons, in WT or *atxr5/*6 (W) two-week-old cotyledon and root tissue. Bar represents mean and whiskers represent +/- standard error (SEM) from *n*=2 biological replicates.

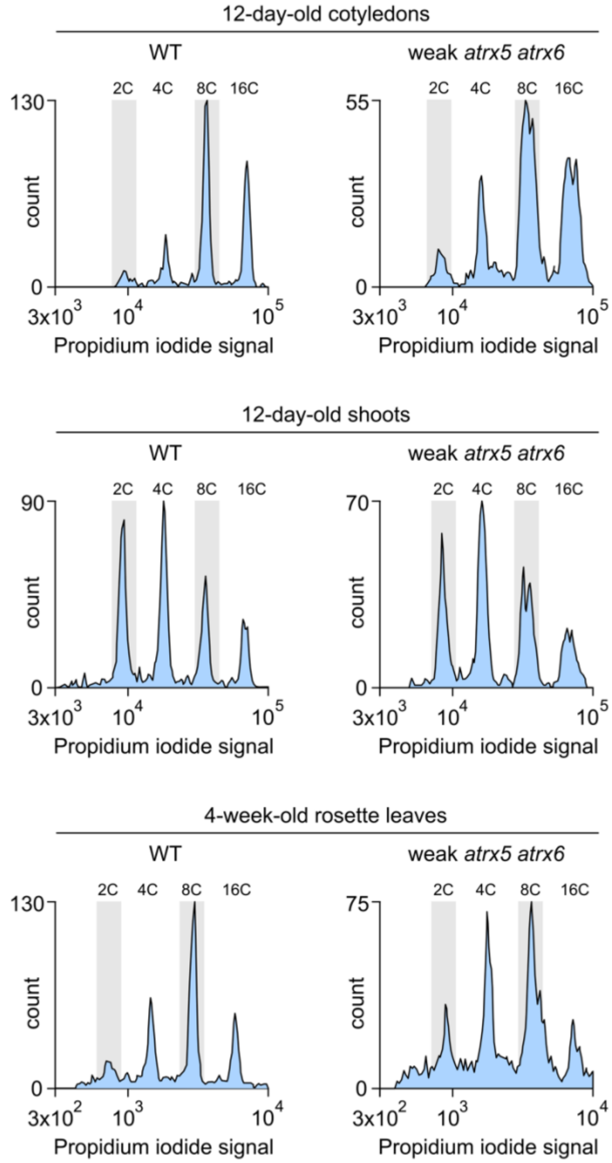

Fig. S2. Flow-cytometry profiles of wild-type and *atxr5/6* (W) mutant plants using 12-day-old cotyledons and shoots and 4-week-old rosette leaves. Nuclei ploidy indicated at top.

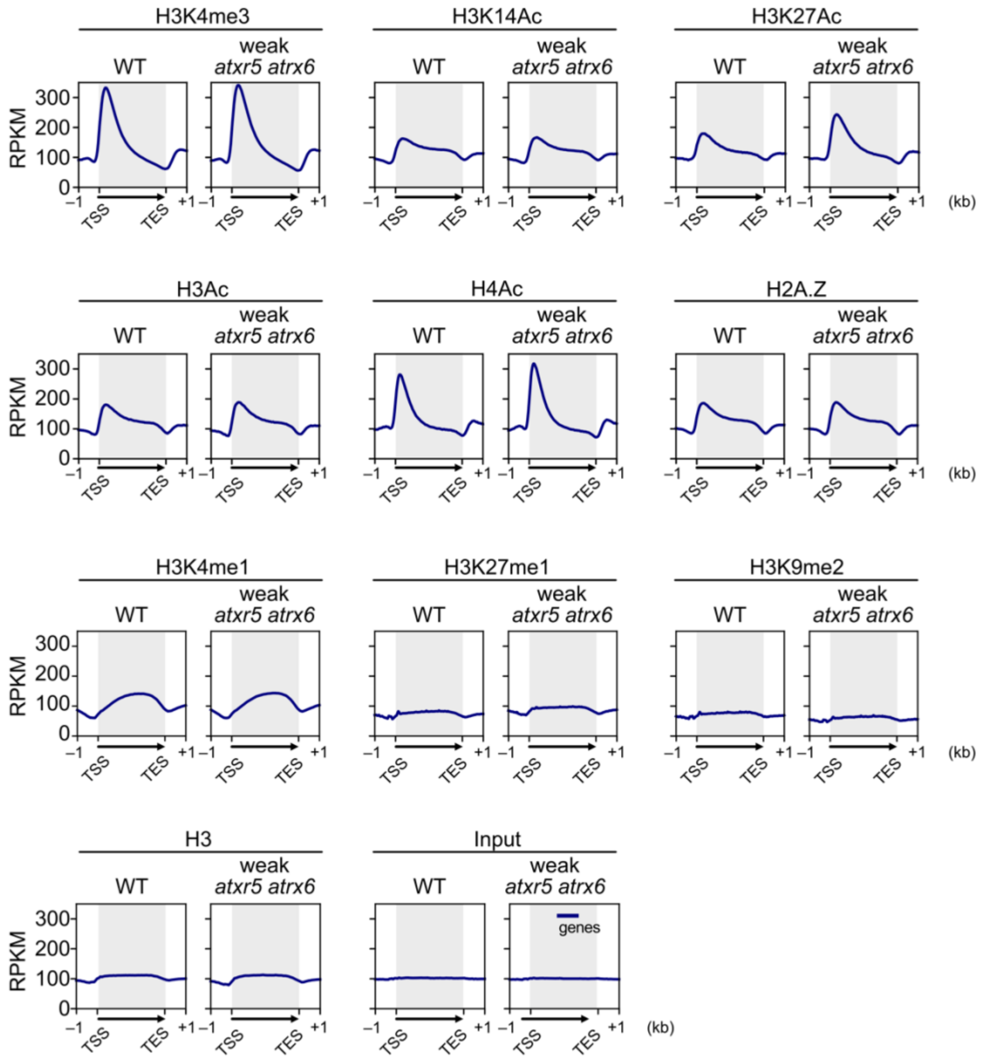

Fig. S3. Distribution of normalized ChIP-seq signal (RPKM) for profiled histone modifications and variants (H3K4me3, H3K14Ac, H3K27Ac, H3Ac, H4Ac, H2A.Z, H3K4me1, H3K27me1, H3K9me2, H3 and input) over protein-coding genes in wild-type and *atxr5/6* (W) cotyledons.

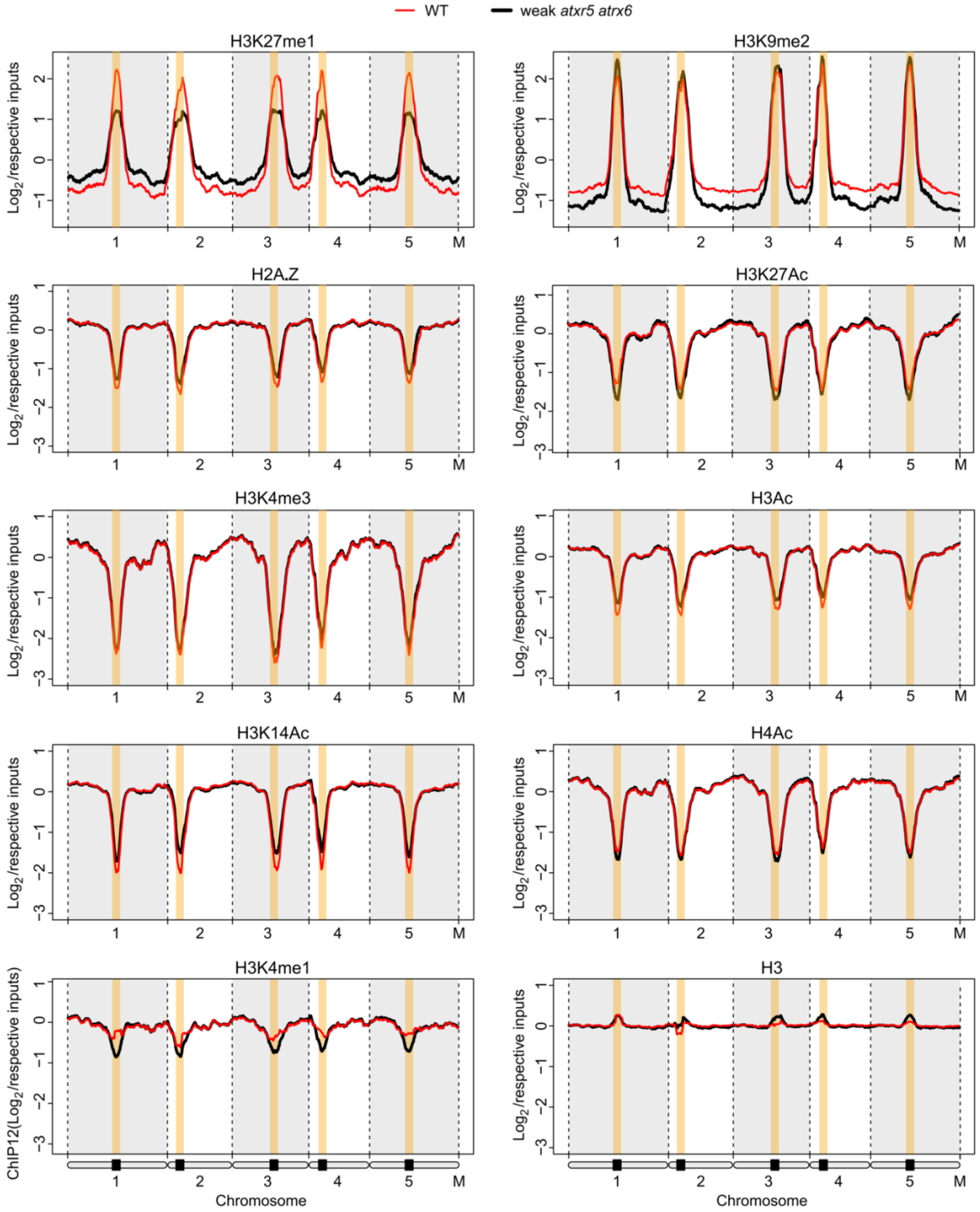

Fig. S4. Whole-genome distribution of 10 profiled histone modifications (H3K4me3, H3K14Ac, H3K27Ac, H3Ac, H4Ac, H2A.Z, H3K4me1, H3K27me1, H3K9me2, H3), plotting smoothed

log2 ratio of normalized signal (RPM) over matched input across 100kb windows in wild-type and *atxr5/6* (W) cotyledons.

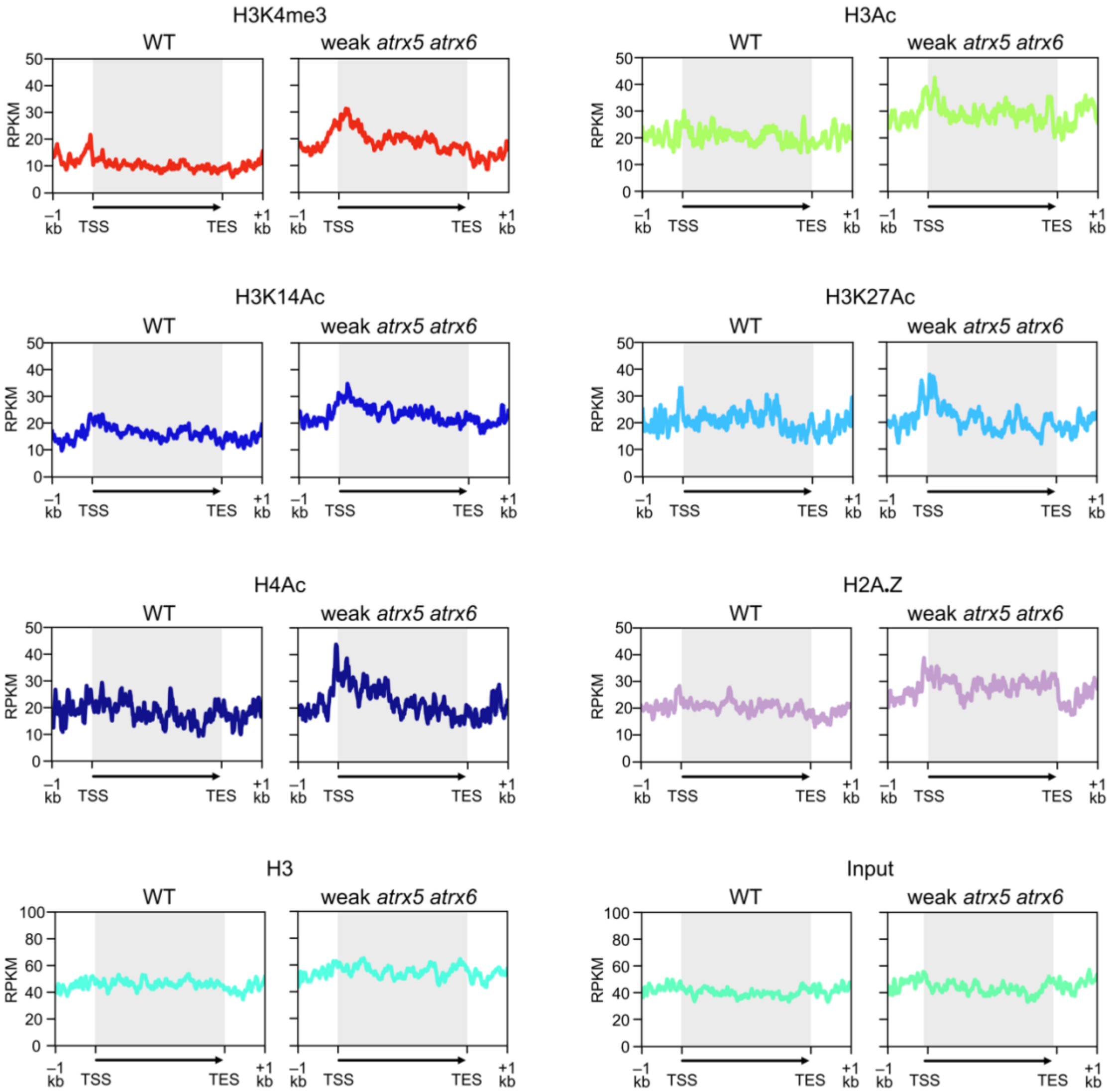

Fig. S5. Distribution of normalized ChIP-seq signal (RPKM) for H3K4me3, H3K14Ac, H3K27Ac, H3Ac, H4Ac, H2A.Z, H3 and input over TEs up-regulated in *atxr5/6* (W), in wild-type and *atxr5/6* (W) cotyledons.

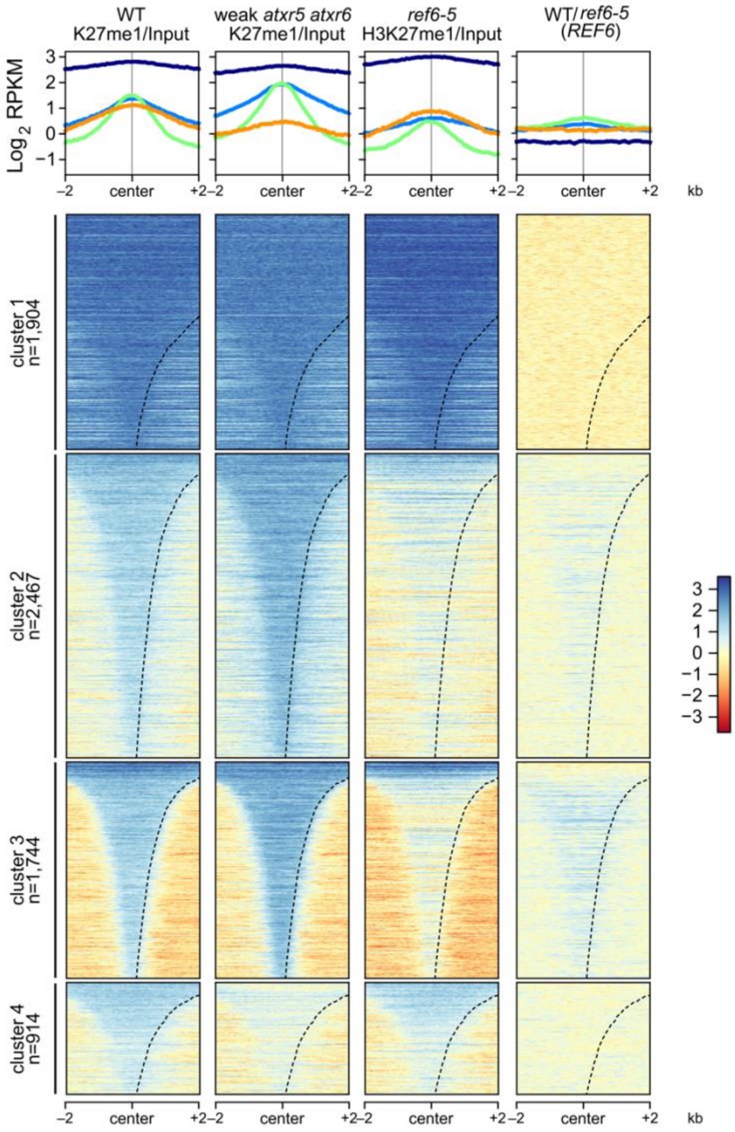

Fig. S6. Profile and heatmap of normalized (log2 RPKM) H3K27me1 signal in WT, *atxr5/6* (W), *ref6-5* and REF6 ChIP-seq over H3K27me1-enriched clusters defined in this study (Fig 2A). Data used with permission from *Antunez-Sanchez et al, 2020*[2], which is licensed under CC BY 4.0 [link to: https://creativecommons.org/licenses/by/4.0/]

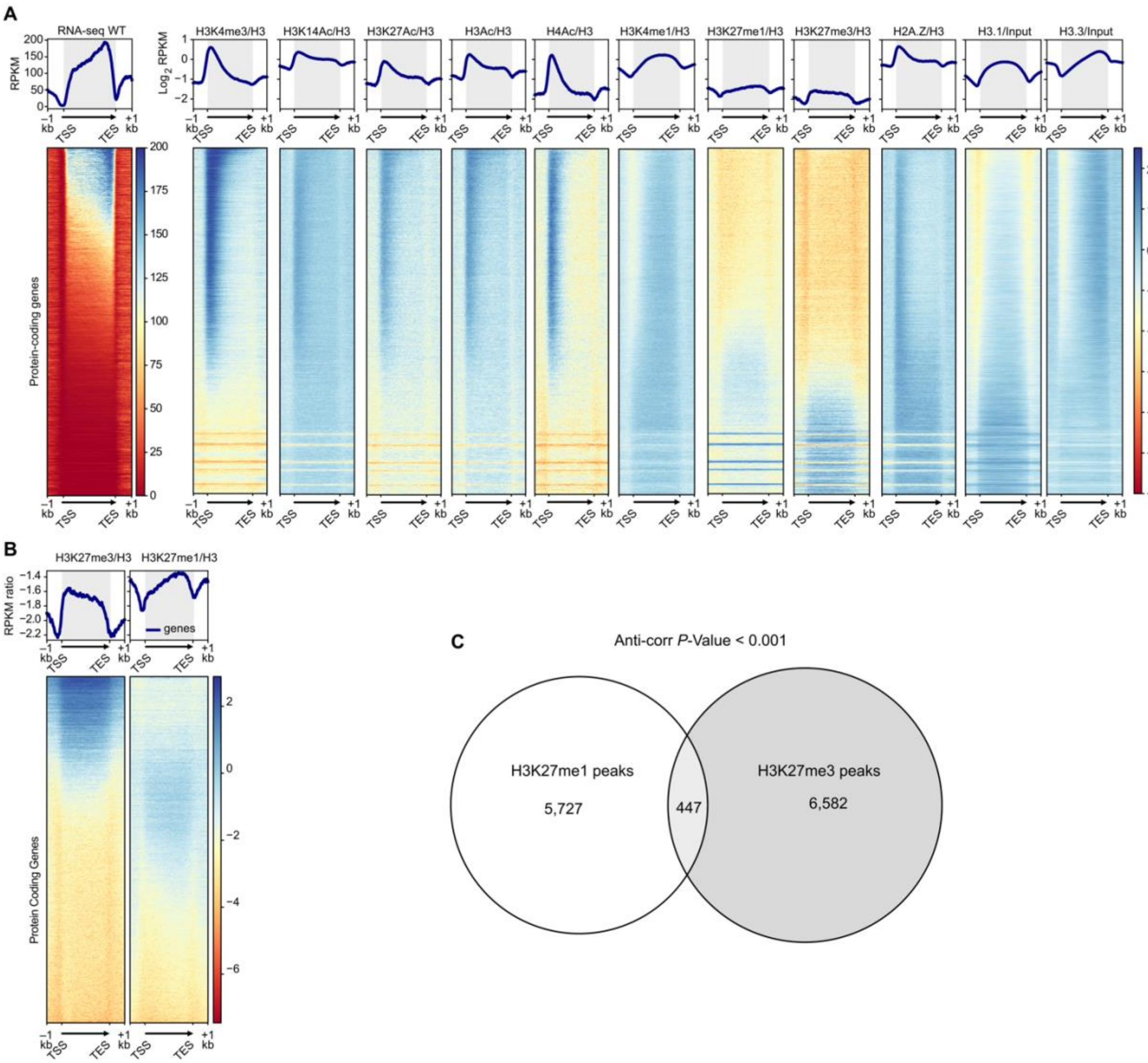

Fig. S7. (A) Distribution of normalized ChIP-seq signal (log2 RPKM) of H3K4me3, H3K14Ac, H3K27Ac, H3Ac, H4Ac, H2A.Z, H3K4me1, H3K27me1, H3K27me3, H3.1 and H3.3 over H3 or input, over protein-coding genes ordered based on gene expression in wild-type cotyledons (four combined replicates) from highest (top) to lowest (bottom). H3.1 and H3.3 data used with permission from *Stroud, et al 2012*[1]**.** **(B)** (Left) Distribution of normalized ChIP-seq signal (RPKM) of H3K27me3 and H3K27me1 over H3 for protein-coding genes ordered based on highest to lowest H3K27me3 signal in WT. **(C)** Overlap between H3K27me3 and H3K27me1 ChIP-seq enriched regions (MACS2, q<0.01).

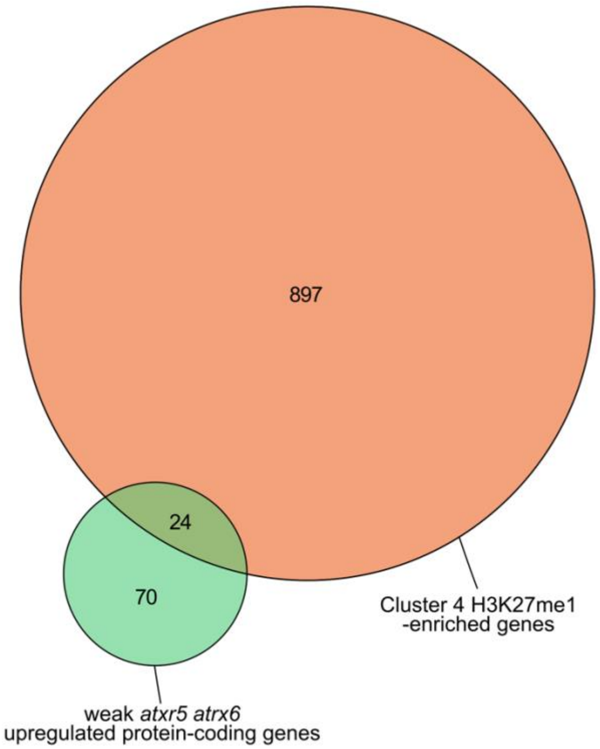

Fig. S8. Overlap between H3K27me1 enriched (MACS2, q<0.01) genes in Cluster 4 and significantly (log 2 fold change >= 1, FDR =< 0.05) up-regulated protein-coding genes in cotyledons.

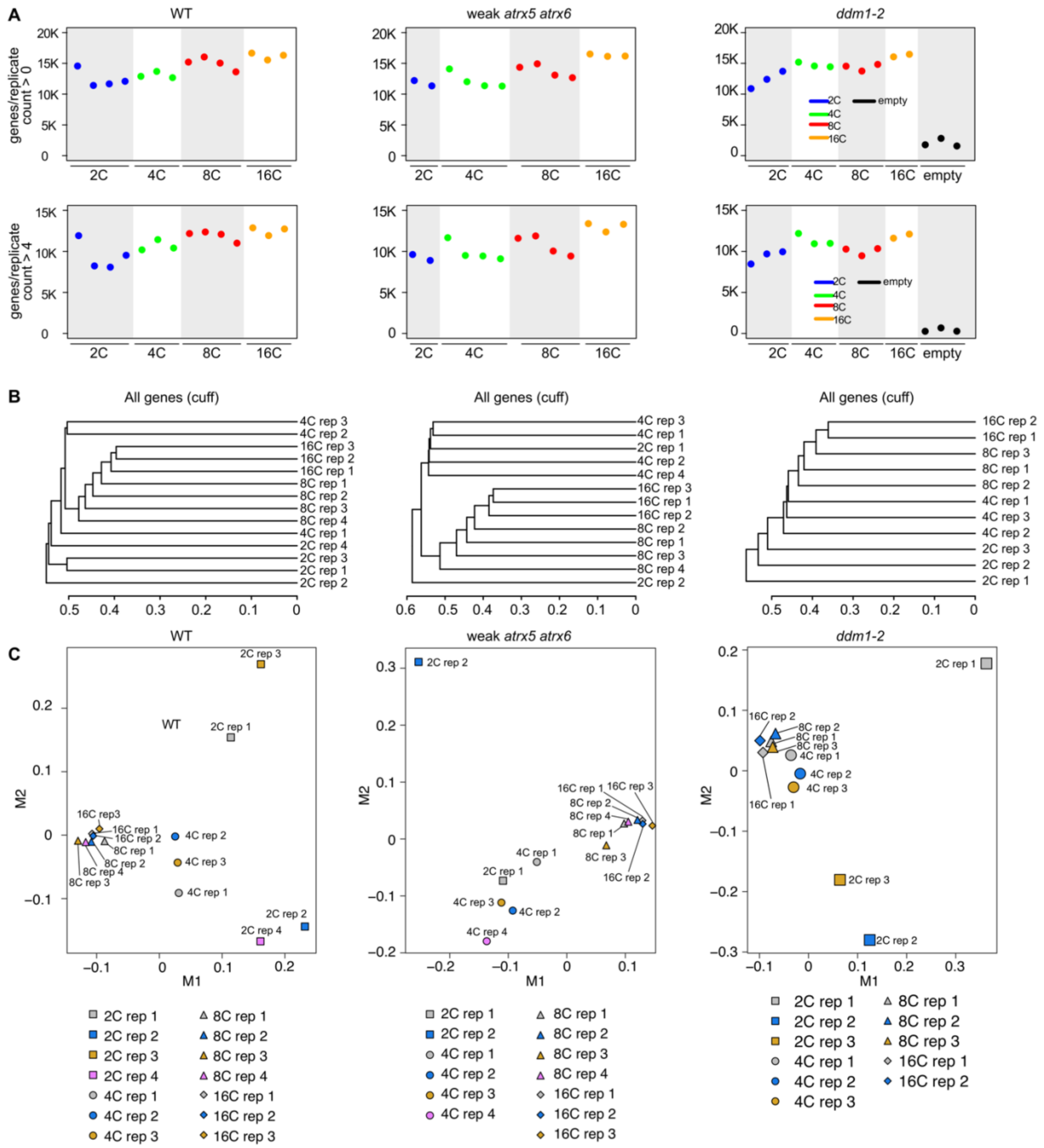

Fig. S9. **(A)** Number of genes detected in Smart-seq2 libraries using a detection threshold of at least one read (top) or five reads (bottom). Each point represents an individual replicate. Rightmost plot also includes three libraries generated from empty wells (no nuclei sorted) as negative controls. **(B)** Dendogram indicating similarity between individual RNA-seq replicates for 2C, 4C, 8C and 16C FACS sorted nuclei in wild-type, *atxr5/6* (W) and *ddm1-2*.

(C) Multi-dimensional scaling (MDS) plots of replicate RNA-seq libraries for 2C, 4C, 8C and 16C FACS sorted nuclei in wild-type, *atxr5/6* (W) and *ddm1-2*.

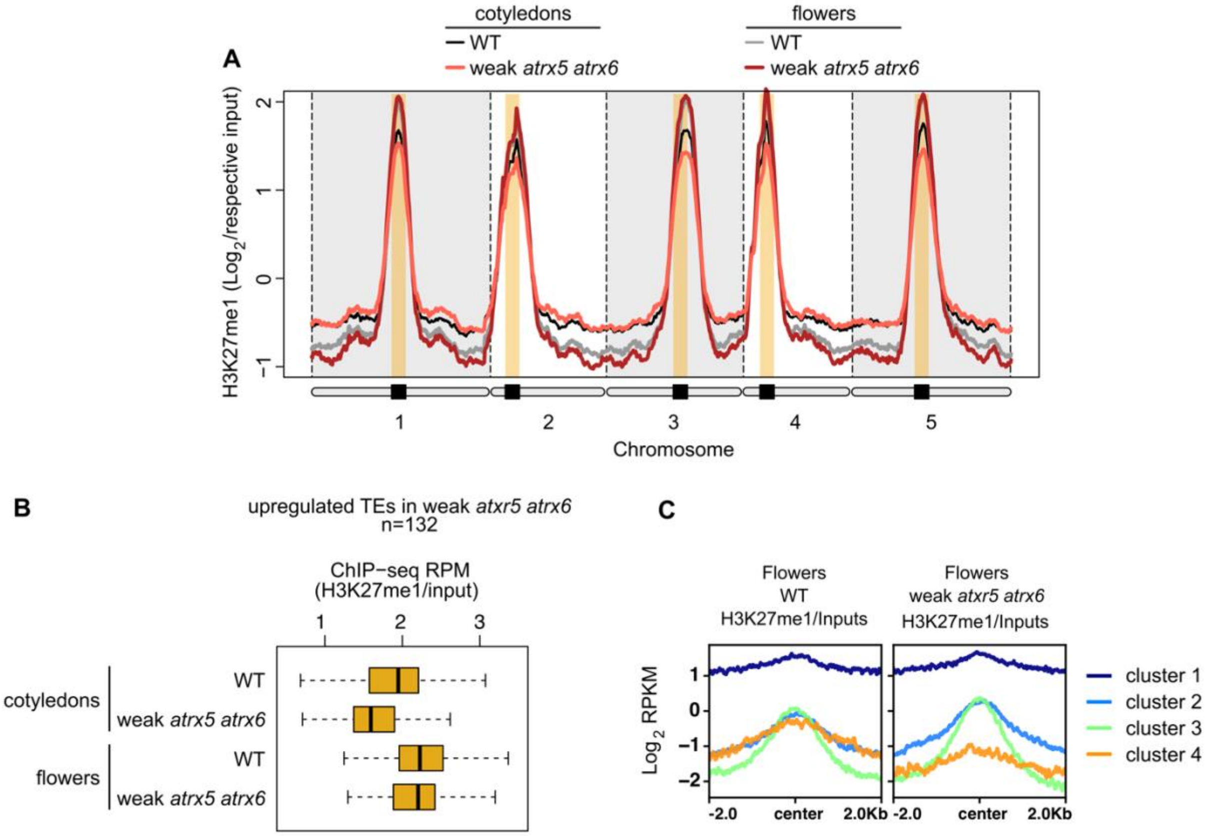

Fig. S10. Characterization of H3K27me1 distribution in wild-type and *atxr5/6* (W) in floral tissue. (A) Whole-genome distribution of H3K27me1 ChIP-seq signal normalized by respective input in wild-type and *atxr5/6* (W) cotyledons and flowers. Smoothed log2 ratio of normalized (RPM) ChIP-seq signal over 100kb windows shown. **(B)** Boxplot of input-normalized H3K27me1 ChIP-seq signal in wild-type and *atxr5/6* (W) cotyledons and flowers, over TEs upregulated in *atxr5/6* (W) in cotyledons. Center line indicates the median, upper and lower bounds represent the 75th and 25th percentile, respectively, whiskers indicate the minimum and the maximum, outliers not shown. **(C)** Profile of input-normalized H3K27me1 signal (log2 RPKM) over the four clusters (Fig. 2A).

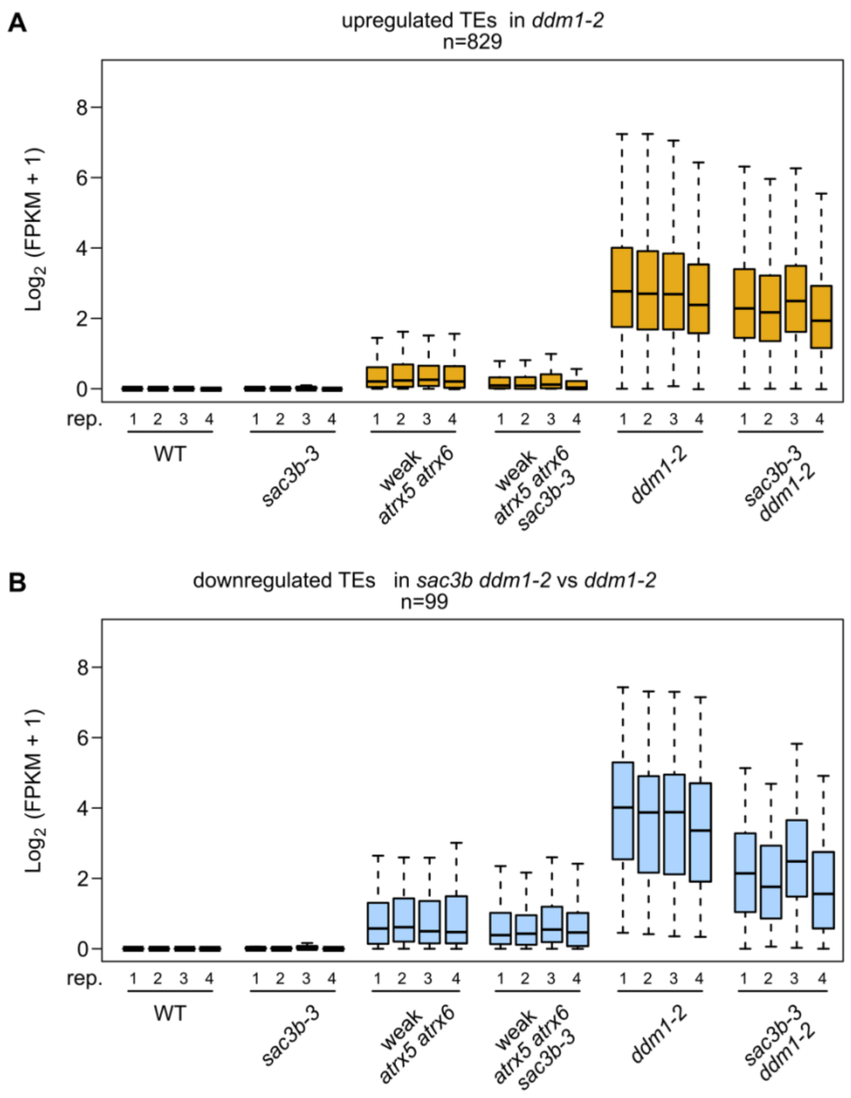

Fig. S11. Boxplot of RNA-seq log2 expression (avg. FPKM+1) of (A) TEs upregulated in *ddm1-2* vs wild-type and (B) TEs down-regulated in *sac3b ddm1-2 vs ddm1-2* in cotyledons, across indicated genotypes and replicates. Center line indicates the median, upper and lower bounds represent the 75th and 25th percentile, respectively, whiskers indicate the minimum and the maximum, and outliers are not plotted.

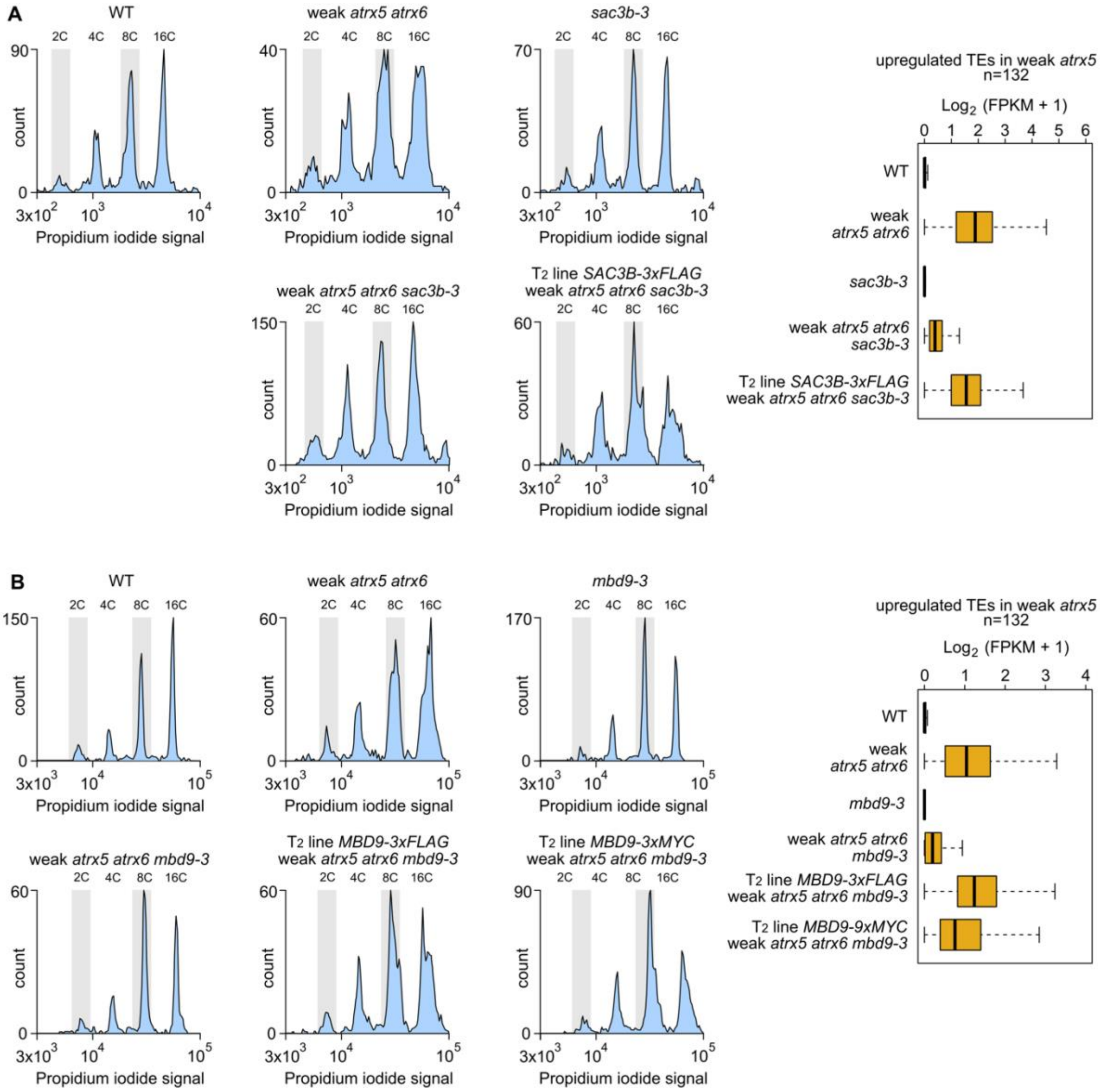

Fig. S12. SAC3B and MBD9 complementation data. (A) Complementation of *sac3b-3 atxr5/6* (W) phenotype. Flow-cytometry profiles of wild-type, *atxr5/6* (W) and T2 *sac3b-3 atxr5/6* (W) + *SAC3B:SAC3B-3XFLAG.* Boxplot of average expression in cotyledons of indicated genotypes over TEs upregulated in *atxr5/6* (W). Center line indicates the median, upper and lower bounds represent the 75th and 25th percentile, respectively, whiskers indicate the minimum and the maximum, outliers not shown. Values plotted represent *n*=1 independent replicates. **(B)** Complementation of the *mbd9-2 atxr5/6* (W) phenotype. Flow-cytometry

profiles of wild-type, *atxr5/6* (W), T2 *mbd9-3 atxr5/6* (W) + *MBD9:MBD9-3XFLAG* and T2 *mbd9-3 atxr5/6* (W) + *MBD9:MBD9-9XMYC.* Boxplot of average expression in cotyledons of indicated genotypes over TEs upregulated in *atxr5/6* (W). Center line indicates the median, upper and lower bounds represent the 75th and 25th percentile, respectively, whiskers indicate the minimum and the maximum, outliers not shown. Values plotted represent *n*=1 independent replicates.

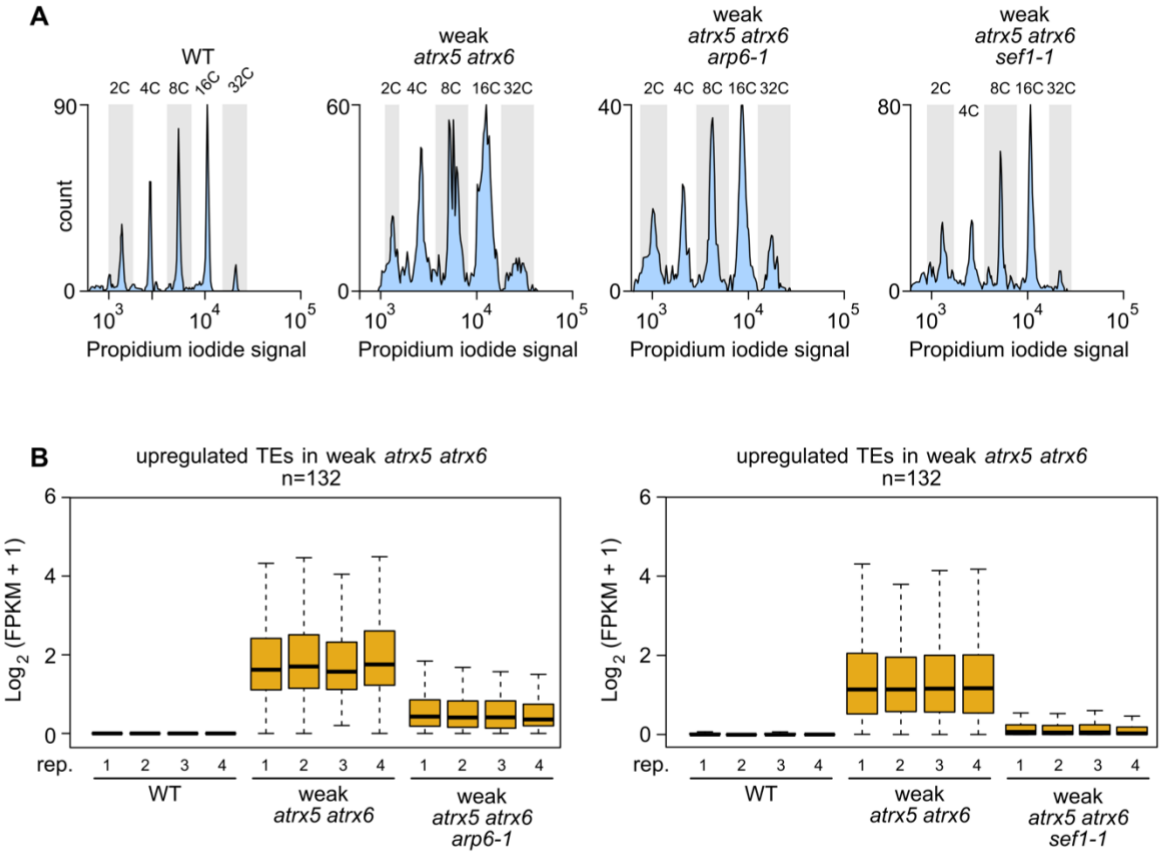

Fig. S13. Conserved components of the SWR1 complex suppress the *atxr5/6* (W) phenotype. (A) Flow-cytometry profiles of wild-type, *atxr5/6, arp6-1 atxr5/6* (W) *and sef1-1 atxr5/6* (W*)* in cotyledons*.* **(B)** Boxplot of average normalized expression in cotyledons of indicated genotypes and replicates over TEs upregulated in *atxr5/6* (W). Center line indicates the median, upper and lower bounds represent the 75th and 25th percentile, respectively, whiskers indicate the minimum and the maximum, and outliers are not plotted.

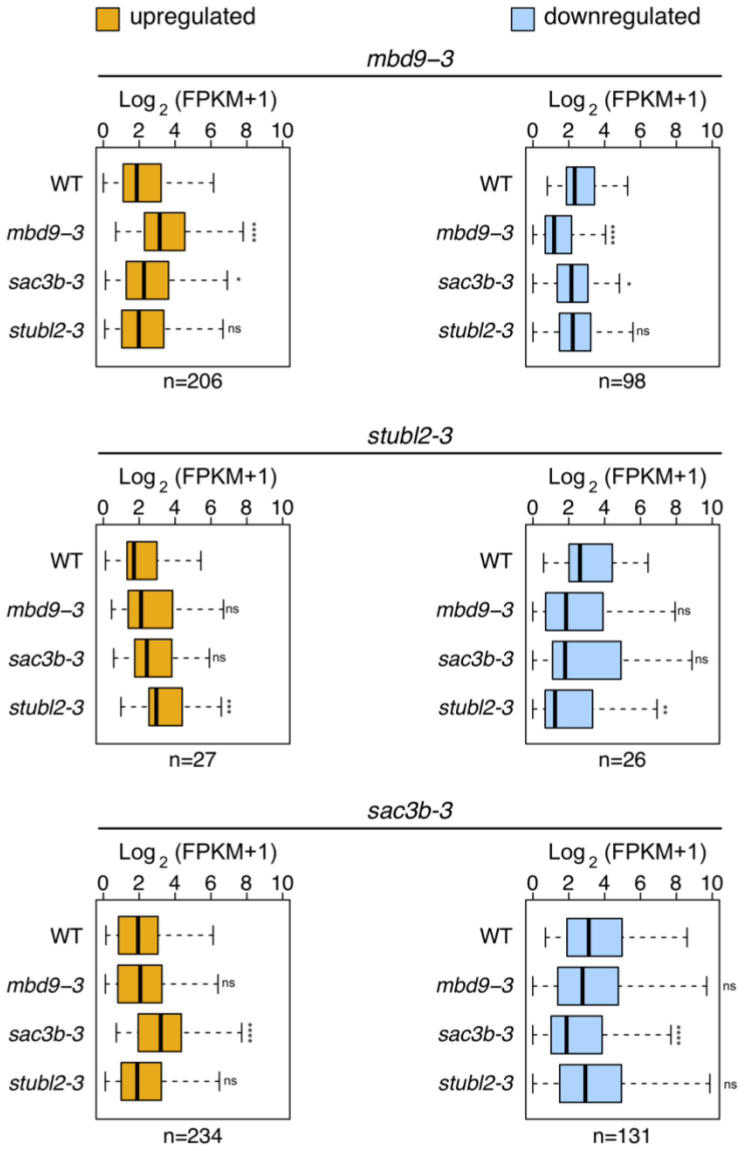

Fig. S14. Boxplot of RNA-seq log2 expression (avg. FPKM+1) for significantly (log2 fold change >= 1, FDR =< 0.05) up-regulated and down-regulated genes for each mutant vs wild-type in cotyledons from *n*=3 biological replicates. Center line indicates the median, upper and lower bounds represent the 75th and 25th percentile, respectively, whiskers indicate the minimum and the maximum, and outliers are not plotted. Unpaired two-sample Wilcoxon test was used to test for significant difference in average expression in wild type vs indicated samples: ns

p-value > 0.05, * p-value <= 0.05, ** p-value <= 0.01, *** p-value <= 0.001, **** p-value <= 0.0001.

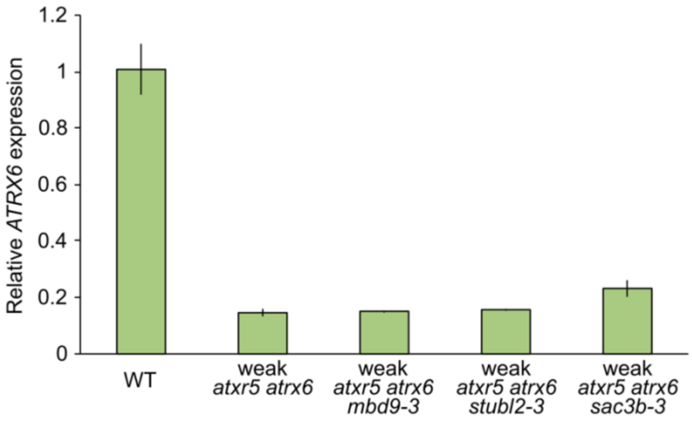

Fig. S15. RT-qPCR analysis of *ATXR6* expression in wild-type, *atxr5/6* (W), *stubl2-3 atxr5/6* (W), *mbd9-3 atxr5/6* (W) and *sac3b-3 atxr5/6* (W) seedlings. Bar represents mean and whiskers represent +/- standard error (SEM) from *n*=3 biological replicates.

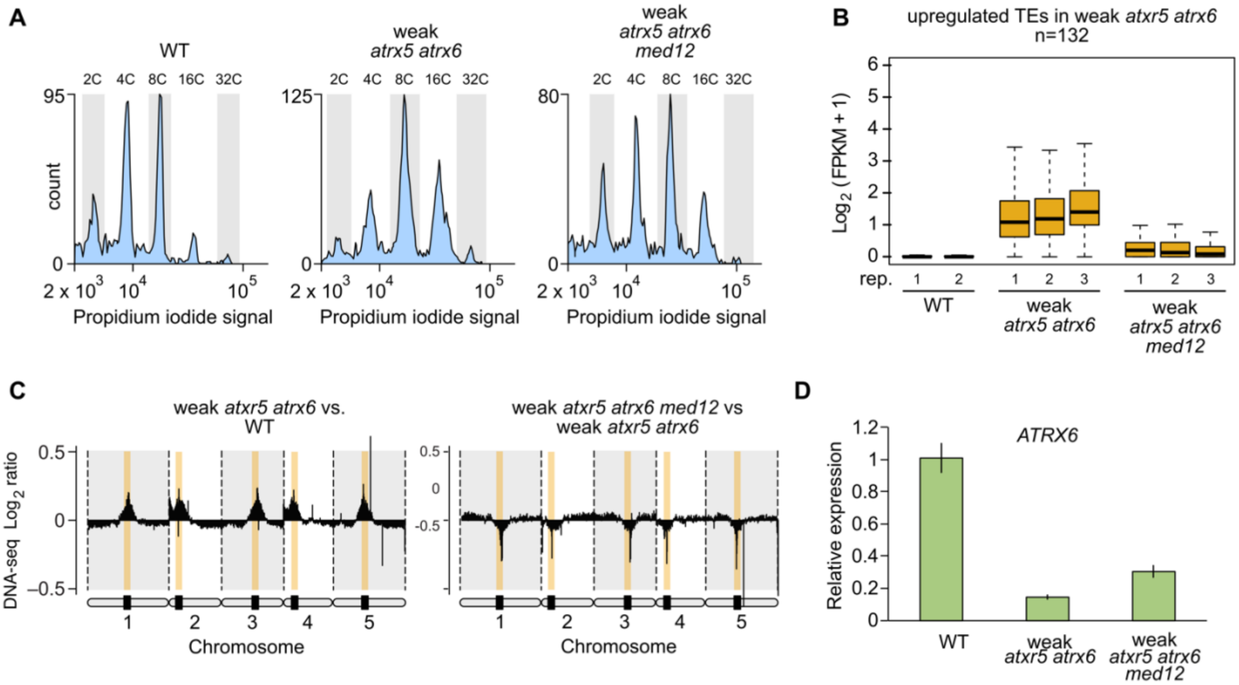

Fig. S16. ***med12* suppresses *atxr5/6* (W) phenotypes. (A)** Flow-cytometry profiles of nuclei from one-month-old leaves of wild-type, *atxr5/6* (W*), med12 atxr5/6* (W) plants*.* **(B)** Boxplot

of RNA-seq log2 expression (avg. FPKM+1) of TEs upregulated in *atxr5/6* (W) cotyledons, in one-month-old leaves of indicated genotypes and replicates. Center line indicates the median, upper and lower bounds represent the 75th and 25th percentile, respectively, whiskers indicate the minimum and the maximum, and outliers are not plotted. **(C)** DNA-Seq of FACS sorted 16C nuclei from one-month-old leaves of wild-type, *atxr5/6* (W*),* and *med12 atxr5/6* (W) plants. Log2 ratio of RPM over 100kb windows. **(D)** RT-qPCR analysis of *ATXR6* expression in wild-type, *atxr5/6* (W), *med12 atxr5/6* (W) seedlings. Error bar represents standard error (SEM) from *n*=3 biological replicates.

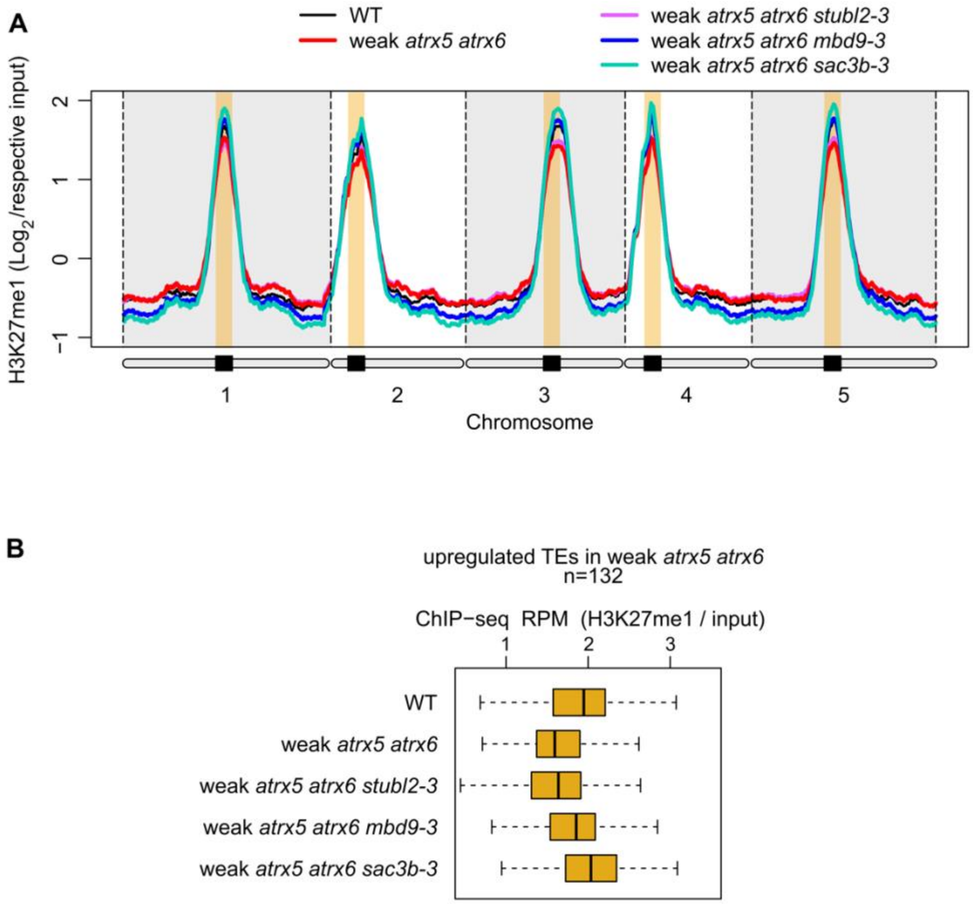

Fig. S17. H3K27me1 ChIP-Seq second replicate for *atxr5/6* (W) suppressors. (A) Whole-genome H3K27me1 ChIP-seq signal in leaves for indicated samples. Smoothed log2 ratio of RPM ChIP-seq signal normalized by input averaged over 100kb windows. **(B)** Boxplot of

RPM-normalized log2(IP/input) H3K27me1 ChIP-seq signal over TEs upregulated in *atxr5/6* (W), for indicated genotypes. Center line indicates the median, upper and lower bounds represent the 75th and 25th percentile, respectively, whiskers indicate the minimum and the maximum, and outliers are not plotted.

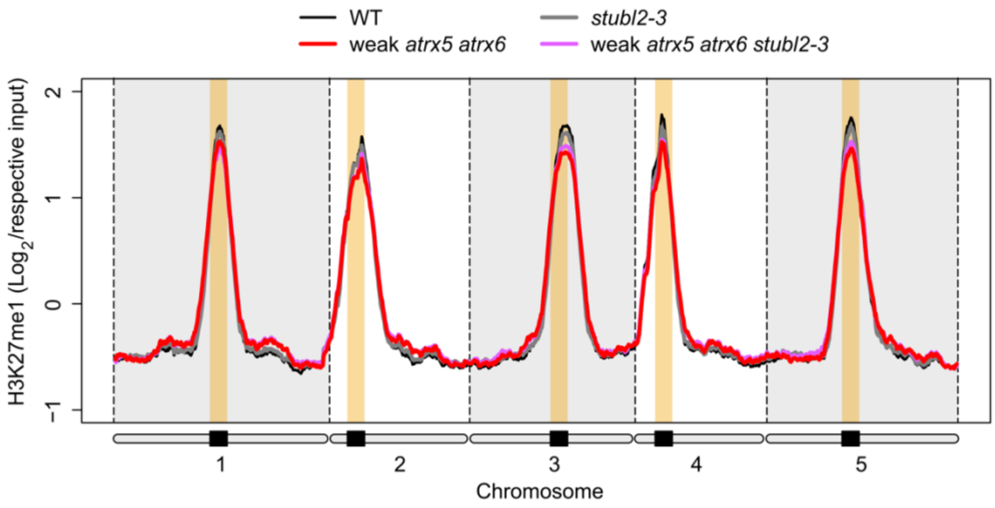

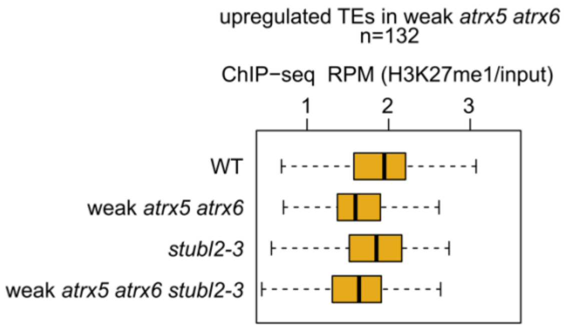

Fig. S18. H3K27me1 in *stubl2-3*. (A) Whole-genome H3K27me1 ChIP-seq signal in leaves for indicated samples. Smoothed log2 ratio of RPM ChIP-seq signal normalized by input averaged over 100kb windows. **(B)** Boxplot of RPM-normalized log2(IP/input) leaf H3K27me1 ChIP-seq signal over TEs upregulated in *atxr5/6* (W) mutant for indicated genotypes. Center line indicates the median, upper and lower bounds represent the 75th and

25th percentile, respectively, whiskers indicate the minimum and the maximum, and outliers are not plotted.

Legends for Supplementary Datasets

Supplementary dataset 1: Raw per-gene read counts from Smart-seq2 libraries generated from 2C, 4C, 8C and 16C sorted nuclei from wild-type, *atxr5/6* (W) and *ddm1-2* cotyledons.

Supplementary dataset 2: IP mass spectrometry. Spectral counts for MBD9-9xMyc, MBD9-3xFlag, SAC3B-3xFlag lines.

Supplementary dataset 3: FPKM expression values for *atxr5/6* related genes in wild-type, *atxr5/6* (W), *stubl2-3 atxr5/6* (W), *mbd9-3 atxr5/6* (W) and *sac3b-3 atxr5/6* (W) cotyledons.

Supplementary dataset 4: List of generated genomic datasets.

SI References

1 Stroud, H. *et al.* Genome-wide analysis of histone H3.1 and H3.3 variants in Arabidopsis thaliana. *Proc Natl Acad Sci U S A* **109**, 5370-5375, doi:10.1073/pnas.1203145109 (2012).

2 Antunez-Sanchez, J. *et al.* A new role for histone demethylases in the maintenance of plant genome integrity. *Elife* **9**, doi:10.7554/eLife.58533 (2020).
